# Analysis and minimization of cellular RNA editing by DNA adenine base editors

**DOI:** 10.1126/sciadv.aax5717

**Published:** 2019-05-08

**Authors:** Holly A. Rees, Christopher Wilson, Jordan L. Doman, David R. Liu

**Affiliations:** 1 Merkin Institute of Transformative Technologies in Healthcare, Broad Institute of Harvard and MIT, Cambridge, MA, USA.; 2 Howard Hughes Medical Institute, Harvard University, Cambridge, MA, USA.; 3 Department of Chemistry and Chemical Biology, Harvard University, Cambridge, MA, USA.

## Abstract

Adenine base editors (ABEs) enable precise and efficient conversion of target A•T base pairs to G•C base pairs in genomic DNA with a minimum of by-products. While ABEs have been reported to exhibit minimal off-target DNA editing, off-target editing of cellular RNA by ABEs has not been examined in depth. Here, we demonstrate that a current ABE generates low but detectable levels of widespread adenosine-to-inosine editing in cellular RNAs. Using structure-guided principles to design mutations in both deaminase domains, we developed new ABE variants that retain their ability to edit DNA efficiently but show greatly reduced RNA editing activity, as well as lower off-target DNA editing activity and reduced indel by-product formation, in three mammalian cell lines. By decoupling DNA and RNA editing activities, these ABE variants increase the precision of adenine base editing by minimizing both RNA and DNA off-target editing activity.

## Teaser

We report cellular RNA editing by adenine base editors and new ABE variants with reduced RNA editing activity.

## INTRODUCTION

Base editors enable the precise installation of targeted point mutations in genomic DNA without creating double-stranded DNA breaks (*1*–*3*). Adenine base editors (ABEs) convert a target A•T base pair to a G•C base pair (*1*). Because the mutation of G•C base pairs to A•T base pairs is the primary form of de novo mutation (*4*), ABEs have the potential to correct almost half of known human pathogenic point mutations (*5*). The original ABE, ABE7.10, can perform remarkably clean and efficient A•T-to-G•C conversion in DNA with very low levels of undesirable by-products such as small insertions or deletions (indels) in cultured cells (*1*, *6*–*8*), adult mice (*8*, *9*), plants (*10*), and other organisms (*11*–*13*). The efficiency of base editors was recently improved through codon and nuclear localization sequence optimization (*6*, *14*) to generate ABEmax (*6*).

Off-target base editing can arise from guide RNA–dependent or guide RNA–independent editing events (*1*, *3*, *15*–*18*). The former results from RNA-guided binding of the Cas9 domain to DNA sites that are similar, but not identical, to the target DNA locus (*7*, *8*, *15*, *18*–*21*). Guide RNA–dependent off-target base editing (*15*, *18*) can be greatly reduced through strategies including installation of mutations that increase DNA specificity into the Cas9 component of base editors (*18*, *20*, *22*), adding 5′-guanosine nucleotides to the single guide RNA (sgRNA) (*18*), or delivery of the base editor as a ribonucleoprotein complex (*18*, *20*, *22*). Guide RNA–independent off-target editing can arise from binding of the deaminase domain of a base editor to C or A bases in a Cas9-independent manner (*3*, *16*, *17*). Recent studies characterized guide RNA–independent off-target DNA editing activity of BE3, the original CBE (cytosine base editor), in mouse embryos (*17*) and in rice (*16*). In contrast to BE3, ABE7.10 in these studies did not result in detectable guide RNA–independent off-target DNA mutations (*16*, *17*).

**Fig. 1:**
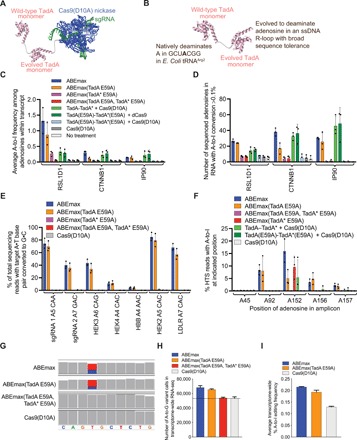
RNA and DNA editing activity of each TadA monomer in ABEmax. (**A**) ABEmax (shown as a schematic model) comprises three proteins fused in a single chain: TadA-TadA*-Cas9(D10A). (**B**) The two TadA monomers (shown as a schematic model) in ABEmax. The schematic models in (A) and (B) are generated from independently solved Cas9 [Protein Data Bank (PDB) id: 4un3] and *E. coli* TadA (PDB id: 1z3a) structures, as the structure of ABE has not yet been solved. (**C**) Average A-to-I conversion frequency in three mRNA transcripts from each treatment analyzed by high-throughput sequencing (HTS). (**D**) The number of adenosines within a 220- to 240-nt region of the indicated mRNA that are converted to inosine [read as a G after cDNA synthesis and DNA sequencing] at a detectable level (≥0.1%). Cas9(D10A) controls show the number of adenosines that are edited by endogenous cellular adenosine deaminases. The amplified regions of RSL1D1, CTNNB1, and IP90 mRNA have 46, 59, and 77 sequenced adenosines, respectively. (**E**) DNA base editing at seven genomic loci from ABEmax or by ABEmax with mutations at catalytic Glu^59^ in TadA or TadA*. The protospacer position of the target A and the sequence context of the A are shown. (**F**) RNA editing frequencies at various adenosines within the RSL1D1 amplicon after treatment with the indicated base editors. The adenosine homologous to TadA’s native substrate is at position 152 within the amplicon. (**G**) On-target DNA base editing with the low-density lipoprotein receptor (LDLR) sgRNA leads to a U-to-C (red to blue) edit in the LDLR mRNA in the transcriptome-wide RNA sequencing (RNA-seq) data. Alignments were visualized in the Integrated Genomics Viewer (IGV) and aligned to hg38. (**H**) Transcriptome-wide RNA-seq analysis showing the number of high-confidence (Phred quality score, ≥20; see Materials and Methods) A-to-I variant calls after treatment with the indicated base editors. The line represents the number of A-to-I conversions in the transcriptome from endogenous deaminase activity as measured in the Cas9(D10A) control samples. (**I**) The average frequency (%) of A-to-I RNA editing across all transcripts. For (A) to (F), data are shown as individual data points and means ± SD for *n* = 3 independent biological replicates performed on different days. For (H) and (I), data are shown as means ± SEM. The alignment was generated by combining reads from three independent biological replicates performed on different days.

All ABEs reported to date are single polypeptide chains containing three fused protein components: a wild-type *Escherichia coli* TadA (tRNA-specific adenosine deaminase) monomer that plays a structural role during base editing, a laboratory-evolved *E. coli* TadA monomer (TadA*) that catalyzes deoxyadenosine deamination, and a Cas9(D10A) nickase (*1*, *3*) (Fig. 1A). *E. coli* TadA natively acts as a homodimer to deaminate an adenosine located in a transfer RNA (tRNA) anticodon loop (*23*), generating inosine (I). We hypothesized that the wild-type TadA monomer, which natively acts on RNA but has strict sequence requirements (*23*, *24*), and/or the evolved TadA* monomer, which we evolved to accept single-stranded DNA (ssDNA) as a substrate and to have broad sequence compatibility, may be able to catalyze the deamination of cellular RNA (Fig. 1B) (*1*, *3*). While we previously observed no substantial ABE7.10-mediated adenosine-to-inosine (A-to-I) editing in human embryonic kidney (HEK) 293T cells among a handful of abundant transcripts sequenced at modest depth (*1*), the association of elevated endogenous A-to-I editing in the transcriptome with disease (*25*) warranted a more comprehensive examination of possible ABE-mediated RNA editing. In particular, recent studies have identified aberrant A-to-I editing as a mechanism by which tumors can develop a resistance to immune checkpoint blockade (*26*).

In this study, we measured, with high sensitivity, A-to-I editing that can be attributed to overexpression of ABEmax, the most efficient ABE variant reported to date (*6*). We used both targeted deep sequencing of individual abundant mRNA transcripts, as well as transcriptome-wide RNA sequencing (RNA-seq), to find that ABEmax can induce low levels of widespread A-to-I editing across the transcriptome. Comparison of RNA editing rates between ABEmax mutants with catalytically disabled deaminase domains revealed that both the wild-type TadA monomer and the evolved TadA* monomer contribute to RNA editing. Guided by an analysis of the TadA structure and the evolved TadA* mutations, we designed a series of ABEmax mutants to minimize RNA editing activity. We found ABEmax variants with mutations in both TadA domains that greatly reduce RNA editing while maintaining efficient target DNA editing, improving DNA specificity, and reducing indel by-product formation. These new ABEmax variants may be especially useful for applications that demand minimal RNA editing and high DNA specificity.

## RESULTS

We began by transfecting HEK293T cells with a plasmid expressing ABEmax and isolating genomic DNA and RNA after 48 hours. After complementary DNA (cDNA) generation from polyadenylated cellular mRNA, we performed high-throughput sequencing (HTS) on 220- to 250-nt (nucleotide) regions of three mRNA amplicons: beta catenin-1 (*CTNNB1*), calnexin (*IP90*), and ribosomal L1 domain containing 1 (*RSL1D1*). We chose *CTNNB1* and *IP90* as two examples of abundant mRNAs in HEK293T cells, and we studied *RSL1D1* because it contains a region highly homologous to the 20-nt region of *E. coli* tRNA^Arg2^ that is the native substrate of TadA (*24*). The TadA minimal substrate sequence is GCUCGGCU**A**CGAACCGAG, while the homologous region of *RSL1D1* mRNA is agUCGGCU**A**CGGAAuuuAG, where uppercase letters indicate sequence identity. In all three transcripts, ABEmax generated low but detectable levels of RNA editing above the endogenous level of A-to-I editing from cellular deaminases (*25*, *27*), which we measured using a Cas9(D10A)-only control. ABEmax expression increased both the extent of A-to-I conversions throughout the transcript (Fig. 1C), measured by the number of sequenced adenosines with an A-to-I conversion frequency of >0.10%, and the magnitude of A-to-I editing (Fig. 1D), measured by the average percentage of A-to-I conversion at every sequenced adenosine. For example, ABEmax generated an average of 1.3 ± 0.41% A-to-I conversion among all sequenced adenosines in RSL1D1 mRNA, a 22-fold increase relative to the Cas9(D10A) nickase–only control that averaged 0.060 ± 0.010% A-to-I conversion in the same transcript. Likewise, ABEmax resulted in detectable deamination of 27 ± 2 of 46 adenosines sequenced in RSL1D1 mRNA, while the Cas9(D10A) nickase–only control resulted in detectable deamination of 7 ± 1 (3.9-fold fewer) of these 46 adenosines (Fig. 1, C and D). To test whether RNA editing activity requires fusion with the Cas9 component of the base editor, we overexpressed the TadA-TadA* monomer in trans with Cas9(D10A) nickase or dead Cas9 and observed substantial RNA editing under these conditions at all three tested transcripts (Fig. 1, C and D). This outcome confirmed that RNA editing activity arises from the unassisted binding of TadA domains to cellular RNA and focused our efforts to improve the DNA:RNA specificity of ABE on engineering these deaminases. Together, these results establish that the TadA-TadA* deaminase component of ABEmax mediates low levels of cellular RNA editing.

Glu^70^ is a critical catalytic residue in *E. coli* TadA, and the TadA E70A mutant either alone (*24*) or in ABE (*1*) has no deaminase activity. In the soluble, N-terminally truncated version of TadA (*24*) used in ABE (*1*), Glu^70^ corresponds to Glu^59^ and will be referred to as Glu^59^ hereafter. To identify which TadA monomers mediate RNA editing in ABEmax, we introduced inactivating E59A mutations into either the TadA or TadA* monomer of ABEmax and measured RNA (Fig. 1, C and D) and DNA (Fig. 1E) editing activity of the resulting variants. Installing the E59A mutation in the wild-type TadA monomer to generate ABEmax(TadA E59A) modestly reduced the average number of edited adenosines in all three tested transcripts relative to ABEmax (Fig. 1C). Despite the modest reduction in RNA editing activity associated with ABEmax(TadA E59A), ABEmax(TadA E59A) maintains high DNA base editing activity similar to that of ABEmax. ABEmax averaged 46.6 ± 3.9% DNA editing across the seven endogenous genomic sites tested, chosen because they result in a wide range of ABEmax editing efficiencies (from 85 ± 6.6% to 4.5 ± 0.70%), while ABEmax(TadA E59A) averaged 41.5 ± 5.4% DNA editing at the same sites (Fig. 1E). ABEmax(TadA E59A) also displayed reduced indel formation at these seven genomic sites compared to ABEmax (fig. S1), from a mean of 2.3 ± 0.39% with ABEmax to 1.1 ± 0.24% with ABEmax(TadA E59A). These data suggest that inactivation of the catalytic domain in the wild-type TadA monomer can reduce off-target RNA editing and indel formation without substantially sacrificing on-target DNA editing efficiency.

By contrast, neither ABEmax(TadA* E59A) nor ABEmax(TadA E59A, TadA* E59A) edit RNA (Fig. 1, C and D) or DNA (Fig. 1E), with one notable exception: ABEmax(TadA* E59A), which contains a wild-type TadA monomer but an inactivated evolved TadA* monomer, edits RSL1D1 mRNA at position 152, the adenosine that is highly homologous to that of TadA’s native tRNA^Arg^ substrate (Fig. 1F). Together, these data indicate that both wild-type TadA and TadA* in ABEmax can deaminate RNA in a Cas9-independent manner. This off-target RNA editing activity can be reduced by inactivating the wild-type TadA monomer, but residual RNA editing activity remains from TadA*, which cannot be inactivated without abolishing DNA editing activity (Fig. 1D).

To test whether these findings apply to many different cellular transcripts, we performed transcriptome-wide analysis of HEK293T cells treated with ABEmax, ABEmax(TadA E59A), ABEmax(TadA* E59A), and ABEmax(TadA E59A, TadA* E59A). We transfected cells with plasmids expressing the base editor and a low-density lipoprotein receptor (*LDLR*)–targeting sgRNA. Targeting the base editors to an expressed gene mimics their typical use (*3*) and enables detection of the on-target U-to-C edit in the corresponding *LDLR* mRNA transcript during transcriptome-wide RNA-seq as an internal positive control (Fig. 1G). Since A-to-I editing in cellular mRNA from endogenous deaminases is a common source of natural RNA editing in metazoans (*25*, *27*), we used cells treated with Cas9(D10A) nickase only as a control to identify A-to-I RNA editing levels from endogenous cellular deaminases.

Transcriptome-wide RNA-seq data revealed that, on average, ABEmax overexpression induced 14,959 additional high-confidence A-to-I edits compared to the Cas9 nickase–only control (Fig. 1H). Although ABEmax overexpression adds only 28% more detected A-to-I edits than the 53,334 endogenous cellular A-to-I edits observed in the Cas9 nickase–only control, these additional ABEmax-induced RNA edits were widespread throughout the transcriptome, including 10,335 transcripts not edited in the Cas9 nickase–only control samples. These data confirm that low-level RNA editing is widespread throughout the transcriptome among cells overexpressing ABEmax.

RNA editing across the transcriptome was reduced by inactivating either TadA or TadA* monomers. Catalytically inactivated ABEmax(TadA E59A, TadA* E59A) resulted in 53,917 A-to-I edits, similar to the 53,334 A-to-I edits detected in the Cas9 nickase–only control. ABEmax(TadA E59A) resulted in 12,142 more A-to-I edits than the Cas9 nickase–only control, 19% fewer additional A-to-I edits than the 14,959 mediated by ABEmax (Fig. 1H). The average A-to-I RNA editing frequency across all transcripts was 0.22% for ABEmax, 0.19% for ABEmax(TadA E59A), and 0.13% for Cas9(D10A) nickase only (Fig. 1I). Together, these findings indicate that transcriptome-wide RNA editing is modestly reduced by inactivating the wild-type TadA monomer in ABEmax.

Given the lack of an elucidated structure of ABE or of the *E. coli* TadA homodimer bound to RNA, we used the crystal structure of *Staphylococcus aureus* TadA, which has high sequence homology to *E. coli* TadA (*23*), to guide the design of ABE mutants that further reduce RNA editing. Starting with ABEmax(TadA E59A), the construct with the inactivated wild-type TadA domain that shows reduced RNA editing but maintains strong DNA base editing (Fig. 1, C and H), we installed mutations into the evolved TadA* monomer.

**Fig. 2:**
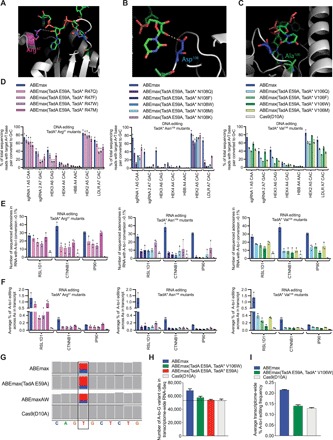
Design and testing of ABEmax mutants with reduced RNA editing activity. Views of the structure of *S. aureus* TadA bound to a minimized version of its native substrate (tRNA^Arg2^) (PDB id: 2B3J) (*23*), showing the residues homologous to Arg^47^ (**A**), Asp^108^ (**B**), and Ala^106^ (**C**) in *E. coli* TadA. Asp^108^ is mutated to Asn^108^ in the evolved TadA*, while Ala^106^ is mutated to Val^106^ in TadA* (*1*). (**D**) DNA base editing at seven genomic loci from ABEmax or ABEmax mutants. (**E**) The number of adenosines converted to inosine at a detectable level (>0.1%) within a 220- to 240-nt region of the indicated mRNA by ABEmax or ABEmax mutants. The amplified regions of RSL1D1, CTNNB1, and IP90 mRNA have 46, 59, and 77 sequenced adenosines, respectively. The Cas9(D10A) controls show the number of adenosines that are edited because of endogenous A-to-I editing activity. (**F**) Average A-to-I RNA editing frequencies by ABEmax or ABEmax mutants among 46 adenosines in RSL1D1, 59 in CTNNB1, and 77 in IP90 mRNA transcripts. (**G**) On-target DNA base editing with the LDLR sgRNA leads to a U-to-C edit in the LDLR mRNA in the transcriptome-wide RNA-seq data. Alignments were visualized in the IGV and aligned to hg38. (**H**) Transcriptome-wide RNA-seq analysis showing the number of high-confidence (Phred quality score, ≥20; see Materials and Methods) A-to-I variant calls after treatment with the indicated base editors. The line represents the number of A-to-I conversions in the transcriptome from endogenous deaminase activity as measured in the Cas9(D10A) control samples. (**I**) The average frequency (%) of A-to-I RNA editing across all transcripts. For (C) to (F), data are shown as individual data points and means ± SD for *n* = 3 independent biological replicates performed on different days. For (H) and (I), data are shown as means ± SEM. The alignment was generated by combining reads from three independent biological replicates performed on different days.

We identified three TadA* residues predicted to interact with the RNA substrate as targets for substitutions that might impair TadA*-mediated RNA deamination. We hypothesized that impeding the ability of TadA* to accommodate 2′-hydroxyl groups that are present in RNA, but absent in DNA, by replacing these three amino acids with larger or more hydrophobic residues (Gln, Phe, Trp, or Met) could further improve the DNA versus RNA editing specificity of ABEmax(TadA E59A). Arg^47^ is predicted to form a hydrogen bond with the 2′-hydroxyl group of the substrate adenosine (Fig. 2A). We replaced Arg^47^ in TadA* with Gln, Phe, Trp, or Met in an effort to abrogate this interaction. We also generated a series of ABEmax mutants with TadA* substitutions at either Asn^108^ (Fig. 2B) or Val^106^ (Fig. 2C), two residues that are located close to the catalytic site of TadA and that mutated from Asp^108^ and Ala^106^ during the evolution of TadA* (*1*). Asp^108^ is predicted to directly hydrogen-bond with the 2′-hydroxyl group of the uridine immediately 5′ of the substrate adenosine (Fig. 2B), and replacement of Ala^106^ might fill some of the space that accommodates this uridine, including its 2′-hydroxyl group, with larger and more hydrophobic side chains (Fig. 2C). We replaced Asn^108^ in ABEmax TadA* with Gln, Phe, Trp, Lys, or Met, and Val^106^ in ABEmax TadA* with Gln, Phe, Trp, or Met, in an effort to disrupt the ability of TadA* to accommodate ribonucleotides by eliminating the possibility of forming hydrogen bonds with 2′-hydroxyl groups in RNA or by steric occlusion. We also tested an additional Asn^108^ Lys mutation to provide a polar side chain that is incapable of serving as a hydrogen bond acceptor, assuming protonation at physiological pH.

We transfected HEK293T cells with each of these 13 ABEmax(TadA E59A) mutants and measured the resulting on-target DNA A•T-to-G•C base editing at the seven genomic loci tested (Fig. 2D). We used HTS of regions of *IP90*, *RSL1D1*, and *CTNNB1* cDNAs to rapidly assess the RNA editing activities of these 13 mutants in HEK293T cells (Fig. 2, E and F) before transcriptome-wide RNA-seq analyses.

Replacing TadA* Arg^47^ in ABEmax(TadA E59A) with Gln, Met, Phe, or Trp maintained relatively high DNA base editing efficiency, particularly at sites where the target A is at protospacer position 5 [counting the protospacer-adjacent motif (PAM) as positions 21 to 23]. Average editing efficiencies were reduced from a mean of 47 ± 3.9% for ABEmax(TadA E59A) to a range of 31 to 41% for the four TadA* Arg^47^ variants. Among the four Arg^47^ mutants tested, ABEmax(TadA E59A, TadA* R47M) and ABEmax(TadA E59A, TadA* R47Q), the most efficient variants for DNA base editing, showed little or no reduction in RNA editing activity compared to ABEmax(TadA E59A) (Fig. 2, E and F). The two variants in which Arg^47^ was replaced with larger and more rigid hydrophobic residues, ABEmax(TadA E59A, TadA* R47F) and ABEmax(TadA E59A, TadA* R47W), resulted in up to a twofold reduction in the number of edited adenosines in the interrogated transcripts (Fig. 2E). Unfortunately, this reduction in RNA editing was accompanied by a similar reduction in DNA editing at sgRNAs in which the target A was located at positions other than position 5 in the protospacer (Fig. 2D). These data indicate that replacing Arg^47^ with Phe or Trp impairs both RNA and DNA editing, and replacing this residue with Met or Gln impairs neither DNA nor RNA editing.

Mutation of TadA* Asn^108^ in ABEmax(TadA E59A) generally preserved DNA base editing at sites in which the target A was at protospacer position 5 but greatly reduced DNA editing at other target sites. The most active Asn^108^ mutant, ABEmax(TadA E59A, TadA* N108K), mediated an average of 25 ± 0.2% on-target DNA editing (Fig. 2D), a 1.9-fold reduction compared to ABEmax, but also exhibited the highest levels of RNA editing among the Asn^108^ mutants assayed (Fig. 2, E and F). Mutation of TadA* Asn^108^ in ABEmax(TadA E59A) to Phe, Trp, Gln, or Met greatly reduced RNA editing compared to ABEmax in the three transcripts sequenced at depth to levels statistically indistinguishable from background RNA editing observed in the Cas9(D10A)-only controls (Student’s two-tailed *t* test, *P* > 0.05 for comparisons between number of edited adenosines in each transcript; Fig. 2E). Together, these data indicate that Asn^108^ in TadA* is important for efficient DNA base editing at protospacer positions beyond the most preferred one (position 5) and is also essential for RNA editing. The ABEmax(TadA E59A, TadA* N108Q) or ABEmax(TadA E59A, TadA* N108W) variants may be useful when the target A is at protospacer position 5 and minimizing RNA editing is critical.

Substitution of TadA* Val^106^ in ABEmax(TadA E59A) resulted in variants that exhibited much lower RNA editing while maintaining DNA editing levels similar to those of ABEmax(TadA E59A) and ABEmax. All four Val^106^ mutants mediated effective DNA base editing across the seven genomic loci tested; the most efficient DNA base editor among these mutants was ABEmax(TadA E59A, TadA* V106W), hereafter referred to as ABEmaxAW, which yielded an average of 36 ± 1.4% A•T-to-G•C DNA editing [compared to 41 ± 5.4% for ABEmax(TadA E59A) and 47 ± 3.9% for ABEmax]. ABEmaxAW exhibited both the highest level of DNA base editing and the lowest level of RNA off-target editing among the Val^106^ mutants tested (Fig. 2, E and F). Analysis of the RNA isolated from cells transfected with ABEmaxAW indicated that the number of detectable A-to-I edits among the regions of the three transcripts analyzed was reduced from an average of 94 ± 8 (of 182 total adenosines) for ABEmax to 26 ± 10 for ABEmaxAW, similar to the background of 12 ± 6 for Cas9 nickase alone (Fig. 2E). The average magnitude of A-to-I edits was also greatly reduced in cells treated with ABEmaxAW (an average frequency of 0.068% A-to-I editing among 182 total adenosines) to levels approaching those observed from Cas9 nickase alone (0.041% average), a 7.2-fold reduction compared with the average of 0.49% A-to-I editing of ABEmax (Fig. 2F). These findings establish that ABEmaxAW greatly reduces off-target RNA editing while preserving most of the on-target DNA editing activity of ABEmax.

We tested the applicability of our findings to other mammalian cell types. First, we compared the DNA base editing activities of ABEmax, ABEmax(TadA E59A), ABEmaxAW, and ABEmax(TadA E59A, TadA* N108W) in HeLa cells (fig. S2) and ABEmax and ABEmaxAW in U2OS and K562 cells (fig. S3). DNA base editing efficiencies among unsorted HeLa and U2OS cells were uniformly lower than in HEK293T cells (fig. S2A), possibly due to poorer transfection or nucleofection efficiencies (*6*). The DNA base editing activity of ABEmaxAW relative to ABEmax and ABEmax(TadA E59A), however, generally remained similar in all three cell types (figs. S2A and S3A). Next, we investigated RNA editing frequencies and magnitudes in U2OS and K562 cells, finding once again that, compared to ABEmax, the use of ABEmaxAW greatly reduced RNA editing to levels indistinguishable from those of the Cas9(D10A) control (fig. S3, C and D). Together, these data indicate that ABEmaxAW can mitigate RNA editing in multiple mammalian cell types.

We assessed the effect of longer exposure time to ABEmax or ABEmaxAW in HEK293T cells by harvesting cells 5 days after transfection, instead of 48 hours. This change increased the average DNA base editing associated with both ABEmax and ABEmaxAW by 1.1-fold, to 52 ± 2.7% for ABEmax and 39 ± 1.7% for ABEmaxAW (fig. S4, A and B). Unexpectedly, average RNA editing was reduced compared to the 48-hour treatment; ABEmax yielded an average frequency of 0.29 ± 0.063% A-to-I editing across the 182 adenosines sequenced (compared with 0.49 ± 0.13% at 48 hours). The average frequency of A-to-I mutation with ABEmaxAW after 5 days (0.074 ± 0.014%, 3.9-fold lower than that of ABEmax) remained close to the background frequency associated with Cas9(D10A) nickase alone of 0.051 ± 0.010% (fig. S4, C and D). We speculate that the steady loss (or silencing) of transfected plasmids expressing base editors, coupled with the constant degradation and replenishment of the transcriptome, may result in lower RNA editing rates at longer time points.

These TadA* mutations might further weaken the ability of ABEmax variants to bind off-target DNA sequences that are already more weakly bound by Cas9. To test this possibility, we measured the levels of off-target DNA editing by ABEmax and a subset of the ABEmax variants described above. We used HTS to assess the frequencies of off-target A·T-to-G·C base editing and indel formation at 12 known off-target sites associated with HEK site 2, HEK site 3, and HEK site 4 (figs. S5 to S7) (*28*). Among these 12 off-target sites, 10 had at least one adenosine within the canonical ABE editing window (from protospacer positions 4 to 8) (*1*, *3*). The mean A·T-to-G·C editing efficiency at these 10 candidate off-target loci from ABEmax was 2.1 ± 0.22%, similar to that of ABEmax(TadA E59A) (2.0 ± 0.28%; figs. S5 to S7). Notably, ABEmaxAW generated an average off-target editing frequency of 0.79 ± 0.18%, a 2.5-fold improvement compared to ABEmax(TadA E59A) and a 2.7-fold improvement relative to ABEmax. Collectively, these results indicate that mutations that reduce the tolerance of ABEmax for RNA editing also increase the DNA specificity of base editing, likely by reducing DNA binding interactions that support productive editing of off-target loci.

Notably, ABEmaxAW also generated 3.7-fold fewer indels than ABEmax at the seven on-target DNA loci tested (from an average of 2.3 ± 0.39% for ABEmax to 0.62 ± 0.0069% for ABEmaxAW; fig. S1). The reason for this reduced indel frequency is unclear, but we hypothesize that indel formation may be dependent on the structure or activity of the wild-type TadA monomer. Consistent with this hypothesis, ABEmax(TadA E59A) also shows reduced average indel formation (1.1 ± 0.24%), and ABEmax(TadA* E59A), which cannot perform DNA base editing, induces indels at an elevated frequency of 4.3 ± 0.45% (fig. S1).

To further illuminate the impact of V106W in TadA* on the DNA and RNA editing activities of ABE, we generated and tested two additional ABEmax mutants: ABEmax(TadA E59, TadA* V106W) (ABEmaxEW) and ABEmax(TadA E59Q, TadA* V106W) (ABEmaxQW) (fig. S8). ABEmaxEW displayed slightly higher DNA on-target editing frequencies than ABEmaxAW (fig. S8A) but also greater indel (fig. S8B) and RNA editing frequencies (fig. S8, C and D), confirming that mutation of both the wild-type and the evolved TadA monomers is required for the most effective reduction in RNA editing and indel frequencies. ABEmaxQW performed as well as or slightly better than ABEmaxAW at on-target DNA base editing (fig. S8A) and displayed similarly low levels of off-target RNA editing (fig. S8, C and D). Consistent with our observations that the wild-type TadA monomer plays a role in indel formation (fig. S1), both ABEmaxEW and ABEmaxQW displayed substantially higher indel frequencies than ABEmaxAW (fig. S8B). These comparisons together indicate that both inactivation of the wild-type TadA and mutation of the evolved monomer with V106W are required to minimize off-target RNA editing, and ABEmaxQW may display higher on-target base editing efficiency at some sites than ABEmaxAW but without the consistently lower indel frequencies of ABEmaxAW.

Last, we performed RNA-seq to identify transcriptome-wide A-to-I editing frequencies associated with ABEmaxAW. We confirmed robust on-target DNA editing activity in the RNA-seq samples treated with ABEmax, ABEmax(E59A), and ABEmaxAW by observing substantial U-to-C mutation in the LDLR mRNA, which resulted from base editing the corresponding genomic DNA site directed by the LDLR-targeting sgRNA (Fig. 2G). While the proportion of edited LDLR mRNA reads is reduced in the ABEmaxAW sample (24 of 90, 27%) compared to the ABEmax (37 of 98, 38%) and ABEmax(TadA E59A) (18 of 66, 27%), the numbers of LDLR mRNA transcripts aligned to the reference sequence are low, making precise quantitation challenging. Consistent with the above results analyzing the three test transcripts in depth, ABEmaxAW only slightly elevated the number of A-to-I edits (57,685) beyond those observed in the Cas9(D10A) nickase–only control (53,334). ABEmaxAW thus resulted in substantially fewer transcriptome edits compared to ABEmax or ABEmax(E59A) [10,608 fewer A-to-I edits than ABEmax and 7791 fewer than ABEmax(E59A); Fig. 2, G and H]. We also compared the average A-to-I RNA editing frequency across all transcripts and found that the average of 0.22% A-to-I RNA editing for ABEmax was reduced to 0.14% for ABEmaxAW and to 0.13% for the Cas9(D10A) nickase–only control (Fig. 2I). These findings confirm that ABEmaxAW maintains strong DNA base editing activity while exhibiting much lower transcriptome-wide RNA editing compared to ABEmax.

**Fig. 3:**
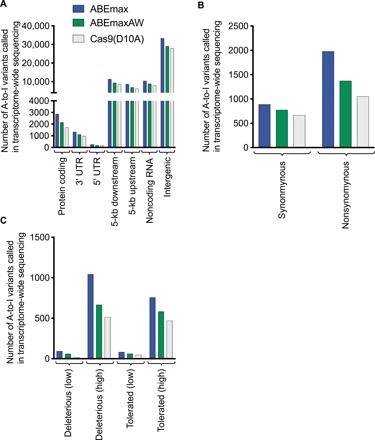
Analysis of A-to-I RNA edits found in transcriptome-wide RNA-seq. (**A**) Classification of the position in which an A-to-I RNA edit was found. “Five-kilobase downstream” refers to mutations that occur within 5-kb downstream of a coding gene, and “5-kb upstream” refers to mutations that occur within the region 5-kb upstream of a coding gene. (**B**) For edits in protein coding regions of mRNAs, edits were classified into synonymous or nonsynonymous mutations. (**C**) For nonsynonymous A-to-I edits in protein-coding regions of RNA, SIFT was used to predict the effect on protein function for these edits. High- or low-confidence calls (indicated in parentheses in the figure) were made according to the standard parameters of the prediction software (see Materials and Methods).

To determine the potential biological significance of the A-to-I edits observed with ABEmax and ABEmaxAW, we used the Ensembl Variant Effect Predictor to determine where the edits were located within mRNA transcripts in our transcriptome-wide sequencing data (Fig. 3A). The RNA editing associated with ABEmax was spread across the transcriptome and not localized to particular regions (fig. S9). Only 4.2% of the A-to-I edits was in a protein-coding region; of these, 69% leads to coding changes (Fig. 3B). Next, we used Sorting Intolerant From Tolerant (SIFT) to predict the impact of these coding changes on protein function, revealing that 58% of the coding A-to-I mutations are predicted to have a deleterious impact on protein function (Fig. 3C). In total, ABEmax induced 1138 A-to-I mutations predicted to be deleterious to protein function, compared to 535 for Cas9(D10A) alone. This was reduced to 727 for ABEmaxAW (Fig. 3C). Mutations in the 3′ or 5′ untranslated region can also be deleterious to protein function, but the effects of these mutations are not readily predictable (*29*). Last, we note that the biological consequence even of mutations that genuinely impair protein function are likely to be minimized by the very low average A-to-I RNA editing frequency of 0.21% for ABEmax, and 0.14% for ABEmaxAW, compared to 0.13% for the Cas9(D10A) nickase–only control.

## DISCUSSION

In this study, we identified widespread, low-level cellular RNA editing from ABEs that was greatly reduced, without substantially sacrificing on-target DNA editing, by introducing the E59A or E59Q mutation into TadA and the V106W mutation in TadA*. In addition to decoupling DNA and RNA editing activities, the ABEmaxAW variant substantially reduced off-target DNA editing activity and the formation of indel by-products. Although we note that even ABEmax-mediated RNA editing is both low level (averaging 0.21% across all transcripts) and transient given the short half-life of most cellular RNAs (*25*, *27*), the extent to which low-level RNA editing may interfere with base editing biological studies or therapeutics development efforts will depend strongly on features of the specific applications, including the duration of exposure to the base editor. We recommend that researchers use ABEmaxAW or ABEmaxQW for adenine base editing applications that require minimizing RNA editing, off-target DNA editing, and/or indel formation.

## MATERIALS AND METHODS

### Plasmid construction

All mammalian cell expression plasmids were constructed by USER cloning from gBlock gene fragments (Integrated DNA Technologies), as previously described (*30*). Phusion U Green Multiplex PCR Master Mix (Thermo Fisher Scientific) was used for amplification of DNA. sgRNA plasmids were constructed by blunt-end ligation of a linear polymerase chain reaction (PCR) product generated by encoding the 20-nt variable protospacer sequence onto the 5′ end of an amplification primer and treating the resulting piece to KLD Enzyme Mix (New England Biolabs) according to the manufacturer’s instruction. Mach1 chemically competent *E. coli* (Thermo Fisher Scientific) cells were used for plasmid construction.

### Mammalian cell culture

All cells were cultured and maintained at 37°C with 5% CO_2_. Antibiotics were not used for cell culture. HEK293T cells [CRL-3216, American Type Culture Collection (ATCC)] and HeLa cells (CCL-2, ATCC) were cultured in Dulbecco’s modified Eagle’s medium plus GlutaMax (Thermo Fisher Scientific) supplemented with 10% (v/v) fetal bovine serum (FBS). K562 cells (CCL-243, ATCC) were cultured in RPMI 1640 medium plus GlutaMax (Thermo Fisher Scientific) supplemented with 10% (v/v) FBS. U2OS cells (HTB-96, ATCC) were cultured in MyCoy’s 5A medium plus GlutaMax (Thermo Fisher Scientific) supplemented with 10% (v/v) FBS.

### Preparation of plasmids for mammalian cell transfection

To obtain endotoxin-free plasmids for transfection, 45 ml of Mach1 cells (Thermo Fisher Scientific) expressing freshly transformed plasmid was pelleted by centrifugation (6000*g* for 5 min at 4°C) and purified using ZymoPURE II Plasmid Midiprep Kits (Zymo Research), according to the manufacturer’s instructions with the inclusion of the optional step of passing the plasmid across the EndoZero Spin Column (Zymo Research). Plasmid yield was quantified using a NanoDrop and by electrophoresis on a 1% agarose tris/borate/EDTA gel supplemented with ethidium bromide.

### Mammalian cell lipofection and genomic DNA isolation

HEK293T cells were seeded on 48-well poly-d-lysine coated plates (Corning) 18 to 20 hours before lipofection. Lipofection was performed at a cell density of 65%. Unless otherwise stated, cells were transfected with 462 ng of nuclease or base editor expression plasmid DNA, 138 ng of sgRNA expression plasmid DNA, and 100 ng of TadA dimer expression plasmid if this was included for “in trans” analysis of RNA editing. Lipofectamine 2000 (1.4 μl; Thermo Fisher Scientific) was used per well. Cells were harvested 48 hours or 5 days, as indicated, after transfection.

HeLa cells were seeded in 250 μl of medium on 48-well collagen-coated plates (Corning) at a density of 70,000 to 80,000 cells/ml 20 to 24 hours before lipofection so cells were approximately 85% confluent at the time of transfection. A total of 200 ng of plasmid was used per well, consisting of a mixture of 154 ng of base editor or Cas9 nickase plasmid and 46 ng of sgRNA expression vector plasmid. 1 μl of HeLaFect (OZ Biosciences) was used per well according to the manufacturer’s instructions. DNA extraction was performed exactly as described above for HEK293T cells.

### Genomic DNA isolation

Medium was removed, and cells were washed once with 1× Dulbecco’s phosphate-buffered saline (Thermo Fisher Scientific). Genomic DNA extraction was performed by addition of 100 μl freshly prepared lysis buffer [10 mM tris-HCl (pH 7.0), 0.05% SDS, and proteinase K (25 μg/ml; Sigma-Aldrich)] directly into the 48-well culture well. The extraction solution was incubated at 37°C for 60 min and then 80°C for 20 min.

### Mammalian cell nucleofection

We combined 560 ng of Cas9(D10A) or base editor expression plasmid with 240 ng of sgRNA-expression plasmid in a volume that did not exceed 1.5 μl. Detailed plasmid maps for plasmids ABEmax and ABEmaxAW are in fig. S10. This combined plasmid mixture was nucleofected in a final volume of 20 μl per sample in a 16-well Nucleocuvette strip (Lonza). K562 cells were nucleofected using the SF Cell Line 4D-Nucleofector X Kit (Lonza) with 5 × 10^5^ cells per sample (program FF-120), according to the manufacturer’s protocol. U2OS cells were nucleofected using the SE Cell Line 4D-Nucleofector X Kit (Lonza) with 3 × 10^5^ to 4 × 10^5^ cells per sample (program DN-100), according to the manufacturer’s protocol. RNA and DNA were isolated 48 hours after nucleofection. U2OS cells were trypsinized and resuspended in phosphate-buffered saline (PBS), and K562 cells were directly resuspended in PBS before being spun down by centrifugation (800*g* for 2 min) to isolate cell pellets. Cell pellets were resuspended in PBS (20 μl), and 3 μl was placed in 50 μl of DNA lysis buffer [10 mM tris-HCl (pH 7.0), 0.05% SDS, and proteinase K (25 μg/ml; Sigma-Aldrich)], which was incubated on a heat block at 37°C for 60 min and then 80°C for 20 min. The remaining 17 μl of cells suspended in PBS was pelleted again by centrifugation (800*g* for 2 min), and RNA extraction was begun on these pellets with the addition of RLT Plus Lysis Buffer (QIAGEN) to the cell pellet. RNA isolation proceeded with the RNeasy Plus Mini Kit (QIAGEN), as described below.

### RNA isolation from mammalian cells

Cells were transfected with the indicated construct, and unless otherwise stated, an sgRNA for the LDLR target site. In the case of HEK293T cells, at the same time, as genomic DNA was harvested from one set of wells that had been transfected with editor in combination with LDLR sgRNA, a second set of wells that had undergone identical treatment were lysed for RNA harvest. RNA isolation was performed with the RNeasy Plus Mini Kit (QIAGEN) according to the manufacturer’s instructions. In short, RNA isolation began with removal of the culture medium and washing of the cells with 1× DBPS (Thermo Fisher Scientific). We added 350 µl of RLT Plus Buffer (QIAGEN) to each well; cells were homogenized by pipetting and transferred into a DNA eliminator column, and the subsequent binding and washing steps for RNA isolation using the RNeasy columns were performed as recommended by the manufacturer. Upon elution of RNA from the RNeasy column with 45 μl of RNase (ribonuclease) free water (QIAGEN), 2 μl of RNaseOUT inhibitor (Thermo Fisher Scientific) was added to prevent RNA degradation, and RNA was stored at −80°C.

### cDNA generation for targeted RNA amplicon sequencing

cDNA generation was performed with SuperScript IV (Thermo Fisher Scientific) according to the manufacturer’s instructions. A poly-T primer was used to selectively amplify mRNAs in the cDNA synthesis step. The optional step of RNase degradation before amplification of cDNAs was included to improve the efficiency of PCR. We note that this step was particularly important for RSL1D1 PCR.

### Preparation of genomic DNA and RNA amplicons for HTS

A two-step PCR protocol was performed as previously reported (*1*). Briefly, 1 μl of isolated genomic DNA was input into the first round of PCR (PCR1). Phusion U Multiplex Master Mix (Thermo Fisher Scientific) was used for both PCR steps. PCR1 was performed with the primers listed in the Supplementary Materials for the appropriate sgRNA treatment for 30 cycles with an annealing temperature of 61°C and an extension time at 72°C for 15 s. Upon verification that PCR1 was successful by running the products on a 2% agarose gel, the barcoding PCR (PCR2) was set up using primers to incorporate barcodes for Illumina sequencing. All primers were ordered from Integrated DNA Technologies. After PCR2, up to 240 samples with different barcode combinations were combined and purified by gel extraction using the QIAquick Gel Extraction Kit (QIAGEN). A second column was used for full removal of agarose and ethidium bromide before the product was quantified using the Qubit ssDNA HS Assay Kit (Thermo Fisher Scientific) and sequenced using an Illumina MiSeq with 220- to 260-bp single-end reads.

For RNA, primers were used as listed in the Supplementary Materials to amplify the targeted region of cDNA. Quantitative PCR (qPCR) was used for all experiments to avoid overamplification of the cDNA. RSL1D1 required more PCR cycles (34) than IP90 and CTNNB1 (32 each) using the program: 98°C for 1 min and 30 s, then cycles of (98°C for 10 s, 60°C for 15 s, and 72°C for 15 s), followed by a final extension of 2 min at 72°C. No reverse transcriptase controls and no input controls were also processed by qPCR and carried forward onto the MiSeq for each experiment. In no instances did either control exceed 2.5% of the number of aligned reads for the particular experiment when compared to the corresponding RNA samples.

For assessing the number of adenosines within an amplicon that showed greater than 0.1% editing, the percentage of G for each adenosine position was measured and counted in Microsoft Excel using the formula = COUNTIF(C85:HS85,“>0.001”), where C85:HS85 represents the range of cells containing the frequency of bases called as a guanosine when the interrogated nucleoside is an adeosnine (for nonadenosine positions, the value within the C85:HS85 range is set to zero).

### Analysis of HTS data for DNA sequencing and targeted amplicon sequencing

Batch analysis with CRISPResso2 (*31*) was used for targeted amplicon and DNA sequencing analysis (*31*). For DNA analysis, a 30-bp window was used to quantify indels around the DNA nick site. Otherwise, the default parameters were used for analysis. The output file “Reference.NUCLEOTIDE_PERCENTAGE_SUMMARY.txt” was imported into Microsoft Excel for quantification of editing frequencies and “CRISPRessoBatch_quantification_of_editing_frequency.txt” for quantification of indel frequencies.

For analysis of RNA amplicon editing, no sgRNA flag was used. Instead, the output file “Reference.NUCLEOTIDE_PERCENTAGE_SUMMARY.txt” was imported into Microsoft Excel for analysis of A-to-G editing rates associated with each sample (inosine in RNA is read as a guanosine by polymerases).

Prism (GraphPad) was used to generate dot plots and bar plots of these data. For instances in the text where means have been calculated across multiple genomic or transcriptomic loci, the SDs reported represent the SD of the mean for all biological replicates.

### Preparation of RNA libraries for RNA-seq

Total RNA was applied to Oligo-dT_25_ Dynabeads (Thermo Fisher Scientific) to enrich for polyadenylated transcripts. Stranded RNA-seq libraries were generated from these samples using the PrepX mRNA 48 Kit (Takara Bio) on the Apollo 324, followed by barcoding and amplification (12 cycles). Following PCR and bead cleanup with AMPure XP beads (Beckman Coulter), libraries were visualized on a 2200 TapeStation (Agilent Technologies) and quantified using the Library Quantification Kit (KAPA Biosystems) for multiplexing. Libraries were sequenced on a NextSeq high-throughput flow cell (Illumina) as 150-bp paired-end reads.

### RNA-seq data analysis

Analysis of the transcriptome-wide editing RNA-seq data was performed as follows. Before the analyses described below, Fastq files were generated using bcl2fastq2 and then trimmed using Trimmomatic version 0.32 to remove adaptor sequences, unpaired sequences, and low-quality bases. We created sam alignments using HISAT2 to align paired reads from each of three biological replicates to the hg38 human reference genome (University of California, Santa Cruz). Precomputed HISAT2 indexes were obtained from https://ccb.jhu.edu/software/hisat2/index.shtml. The resulting sam files were sorted and indexed using the SAMtools software package. Sorted bam alignments from three biological replicates were combined using SAMtools to increase coverage and provide high-quality variant calls. Combined bams were randomly down-sampled to 120 million aligned reads for each condition using a random number generator. The SEM was found by repeated random down-sampling (from the total number of aligned reads to 120 million aligned reads) and measuring the spread in the variant calling results, which arise from different random sampling events.

Variant calling was performed using the freebayes software package version 1.2.0 (https://github.com/ekg/freebayes), an inherently probabilistic measure that accounts for error. The resulting VCF files were filtered with vcftools to retain only A-to-G variants, common variants, and variant calls with a call quality greater than or equal to 20, thus removing sites with less than a 0.99 probability of corresponding to a position where a real A-to-I edit has occurred. Thus, the variant calling performed here considers read depth at a specific adenosine, number of edited reads at that position, mapping quality, and base call quality, and, using all of these indicators, returns the probability that there is bona fide RNA editing at that given adenosine.

### Effect prediction of the A-to-I variants identified by RNA-seq

The Variant Effect Predictor (Ensembl) was used to determine the location within a transcript of each A-to-I edit found in the sample treated with ABEmax, Cas9(D10A), or ABEmaxAW and whether the mutation was synonymous or nonsynonymous. The category “downstream gene variant” includes mutations found within a region 5-kb downstream of the start of a gene, and the category “upstream gene variant” includes mutations found in the region 5-kb upstream of a protein-coding region. “Intergenic regions” includes A-to-I mutations occurring in noncoding regions more than 5 kb away from the beginning or end of a coding region. SIFT (https://sift.bii.a-star.edu.sg/) was used to predict the outcome of nonsynonymous mutations on protein function. High- and low-confidence calls were made using standard SIFT parameters.

### Calculation of the average frequency of A-to-I editing across the transcriptome

To calculate the average frequency of A-to-I RNA editing among adenosines sequenced in transcriptome-wide sequencing analysis, we used REDItools to quantify the percentage of A-to-I editing in each sample (https://github.com/tflati/reditools2.0). We removed all nucleotides except adenosines from our analysis and then removed all adenosines with a read coverage of less than 20 to avoid errors due to low sampling. Next, we calculated the number of adenosines converted to an inosine in each sample and divided this by the total number of adenosines in our dataset after filtering to obtain a percentage of adenosines edited to inosine in the transcriptome. The calculation of SEM was performed as described in the variant calling section.

### Analysis of the transcriptome-wide position of A-to-I edits

The transcriptome-wide RNA-seq data were demultiplexed and aligned as described above. Bins of 1 million nt were created along the human genome using bedtools makewindows. The high-confidence A-to-I edits were counted per bin using bedtools coverage. Last, the data were plotted in R using plot_ly and IdeoViz to show single-nucleotide polymorphism density per bin.

## Supplementary Material

http://advances.sciencemag.org/cgi/content/full/5/5/eaax5717/DC1

Download PDF

## SUPPLEMENTARY MATERIALS

Supplementary material for this article is available at http://advances.sciencemag.org/cgi/content/full/5/5/eaax5717/DC1 Fig. S1. Indel frequencies associated with ABEmax and engineered ABEmax mutants. Fig. S2. DNA base editing and indel formation in HeLa cells from ABEmax and ABEmax mutants. Fig. S3. DNA base editing, indel formation, and RNA editing in U2OS and K562 cells harvested 48 hours after nucleofection with ABEmax, ABEmax mutants, or Cas9(D10A). Fig. S4. DNA base editing, indel formation, and RNA editing in HEK293T cells harvested 5 days after transfection with ABEmax or ABEmax mutants. Fig. S5. Off-target DNA base editing associated with the HEK site 2 locus by ABEmax and ABEmax mutants. Fig. S6. Off-target DNA base editing associated with the HEK site 3 locus by ABEmax and ABEmax mutants. Fig. S7. Off-target DNA base editing associated with the HEK site 4 locus by ABEmax and ABEmax mutants. Fig. S8. DNA base editing, indel formation, and RNA editing in HEK293T cells harvested 48 hours after transfection with ABEmax, ABEmaxAW, ABEmaxQW or ABEmax(TadA* A106V). Fig. S9. A-to-I RNA editing across the transcriptome for ABEmax, ABEmaxAW, ABEmax(TadA E59A), and Cas9(D10A). Fig. S10. Depiction of plasmid maps used in this study. Table S1. Guide RNA sequences. Table S2. Primers used for amplification of genomic DNA or cDNA for HTS. Table S3. List of amplicon sequences used for alignment and analysis of HTS reads. Table S4. List of primers used to amplify genomic off-target loci. Table S5. List of interrogated off-target genomic loci (*28*), with guide RNA sequences and amplicons used for alignment. Table S6. List of plasmid accession numbers from Addgene.
