# Supplementary material for: Analysis and minimization of cellular RNA editing by DNA adenine base editors
Source: Sci Adv. 2019 May 8;5(5):eaax5717. doi: 10.1126/sciadv.aax5717 (PMC6506237; doi:10.1126/sciadv.aax5717)
Supplement: http://advances.sciencemag.org/cgi/content/full/5/5/eaax5717/DC1 [file supp_5_5_eaax5717__index.html]

Science Advances | Science Advances

## Supplementary Materials

**This PDF file includes:**

- Fig. S1. Indel frequencies associated with ABEmax and engineered ABEmax mutants.
- Fig. S2. DNA base editing and indel formation in HeLa cells from ABEmax and ABEmax mutants.
- Fig. S3. DNA base editing, indel formation, and RNA editing in U2OS and K562 cells harvested 48 hours after nucleofection with ABEmax, ABEmax mutants, or Cas9(D10A).
- Fig. S4. DNA base editing, indel formation, and RNA editing in HEK293T cells harvested 5 days after transfection with ABEmax or ABEmax mutants.
- Fig. S5. Off-target DNA base editing associated with the HEK site 2 locus by ABEmax and ABEmax mutants.
- Fig. S6. Off-target DNA base editing associated with the HEK site 3 locus by ABEmax and ABEmax mutants.
- Fig. S7. Off-target DNA base editing associated with the HEK site 4 locus by ABEmax and ABEmax mutants.
- Fig. S8. DNA base editing, indel formation, and RNA editing in HEK293T cells harvested 48 hours after transfection with ABEmax, ABEmaxAW, ABEmaxQW or ABEmax(TadA\* A106V).
- Fig. S9. A-to-I RNA editing across the transcriptome for ABEmax, ABEmaxAW, ABEmax(TadA E59A), and Cas9(D10A).
- Fig. S10. Depiction of plasmid maps used in this study.
- Table S1. Guide RNA sequences.
- Table S2. Primers used for amplification of genomic DNA or cDNA for HTS.
- Table S3. List of amplicon sequences used for alignment and analysis of HTS reads.
- Table S4. List of primers used to amplify genomic off-target loci.
- Table S5. List of interrogated off-target genomic loci (*28*), with guide RNA sequences and amplicons used for alignment.
- Table S6. List of plasmid accession numbers from Addgene.

Download PDF

**Files in this Data Supplement:**

- Adobe PDF - aax5717\_SM.pdf
