# Supplementary material for: Analysis and minimization of cellular RNA editing by DNA adenine base editors
Source: Sci Adv. 2019 May 8;5(5):eaax5717. doi: 10.1126/sciadv.aax5717 (PMC6506237; doi:10.1126/sciadv.aax5717)
Supplement: Download PDF [file aax5717_SM.pdf]

Science Advances
AAAS

advances.sciencemag.org/cgi/content/full/5/5/eaax5717/DC1

Supplementary Materials for

Analysis and minimization of cellular RNA editing by DNA adenine base editors

Holly A. Rees, Christopher Wilson, Jordan L. Doman, David R. Liu*

*Corresponding author. Email: drliu@fas.harvard.edu

Published 8 May 2019, *Sci. Adv.* **5**, eaax5717 (2019)
DOI: 10.1126/sciadv.aax5717

This PDF file includes:

Fig. S1. Indel frequencies associated with ABEmax and engineered ABEmax mutants.
Fig. S2. DNA base editing and indel formation in HeLa cells from ABEmax and ABEmax mutants.
Fig. S3. DNA base editing, indel formation, and RNA editing in U2OS and K562 cells harvested 48 hours after nucleofection with ABEmax, ABEmax mutants, or Cas9(D10A).
Fig. S4. DNA base editing, indel formation, and RNA editing in HEK293T cells harvested 5 days after transfection with ABEmax or ABEmax mutants.
Fig. S5. Off-target DNA base editing associated with the HEK site 2 locus by ABEmax and ABEmax mutants.
Fig. S6. Off-target DNA base editing associated with the HEK site 3 locus by ABEmax and ABEmax mutants.
Fig. S7. Off-target DNA base editing associated with the HEK site 4 locus by ABEmax and ABEmax mutants.
Fig. S8. DNA base editing, indel formation, and RNA editing in HEK293T cells harvested 48 hours after transfection with ABEmax, ABEmaxAW, ABEmaxQW or ABEmax(TadA* A106V).
Fig. S9. A-to-I RNA editing across the transcriptome for ABEmax, ABEmaxAW, ABEmax(TadA E59A), and Cas9(D10A).
Fig. S10. Depiction of plasmid maps used in this study.
Table S1. Guide RNA sequences.
Table S2. Primers used for amplification of genomic DNA or cDNA for HTS.
Table S3. List of amplicon sequences used for alignment and analysis of HTS reads.
Table S4. List of primers used to amplify genomic off-target loci.
Table S5. List of interrogated off-target genomic loci (*28*), with guide RNA sequences and amplicons used for alignment.
Table S6. List of plasmid accession numbers from Addgene.

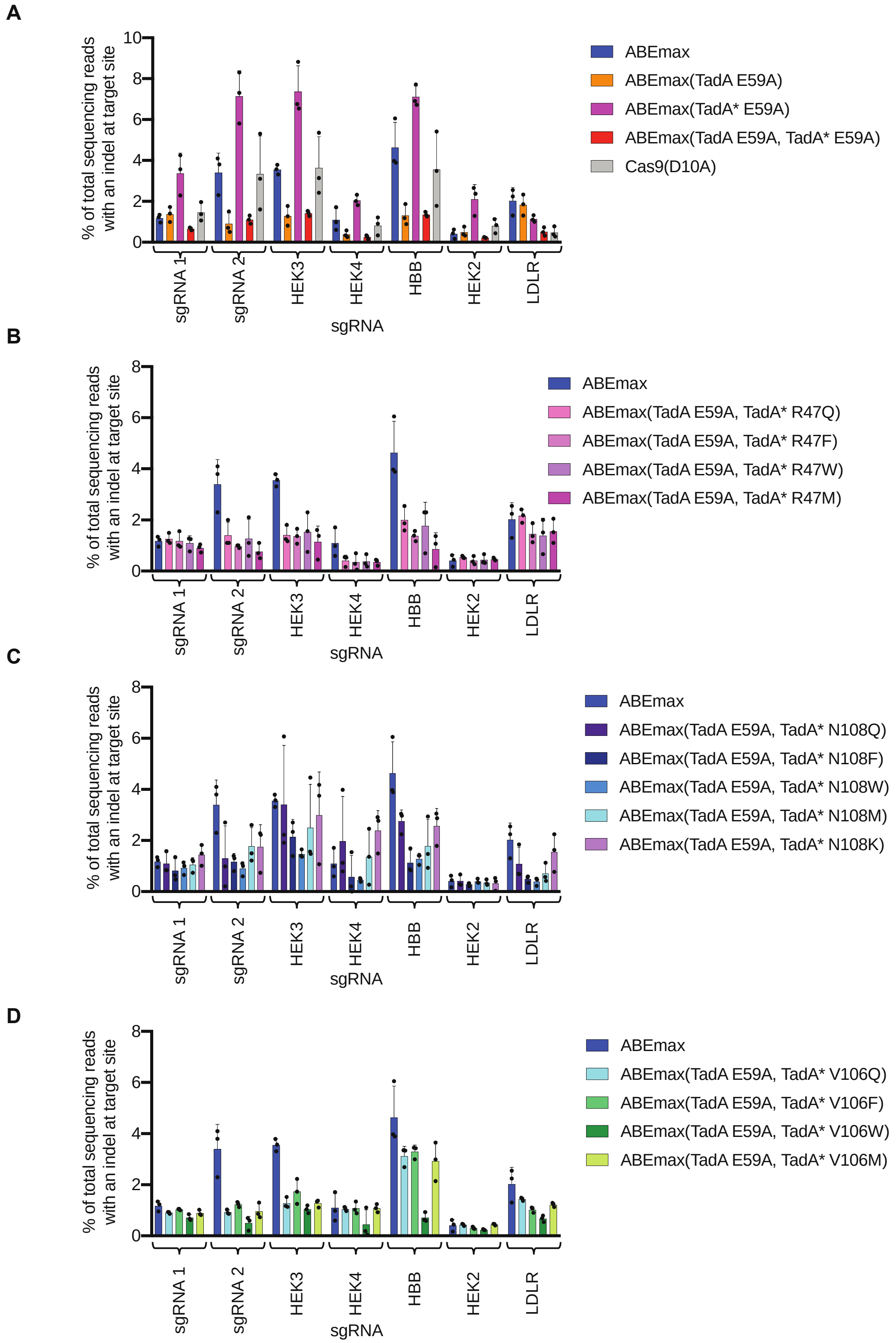

Fig. S1. Indel frequencies associated with ABEmax and engineered ABEmax mutants. (A) Catalytically disabled ABEmax variants. (B) ABEmax(TadA E59A) variants with mutations at Arg 47 in TadA*. (C) ABEmax(TadA E59A) variants with mutations at Asn 108 in TadA*. (D) ABEmax(TadA E59A) variants with mutations at Val 106 in TadA*. Individual data points and mean±s.d. for n=3 independent biological replicates, performed on different days.

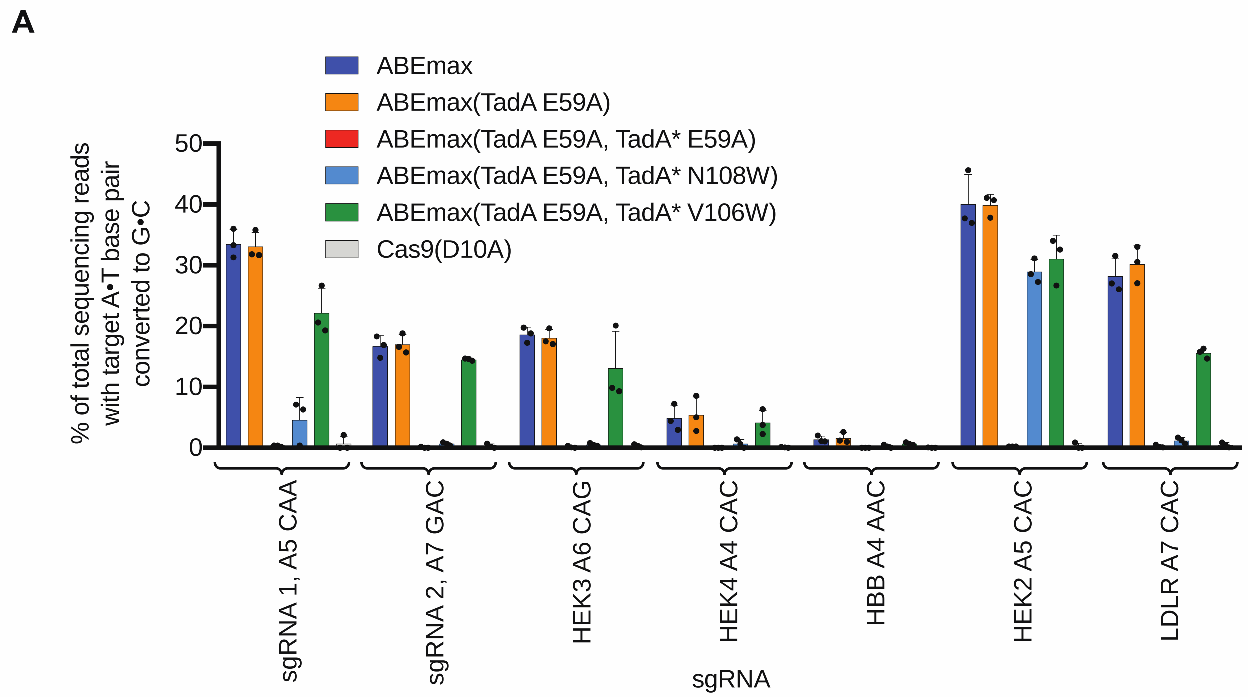

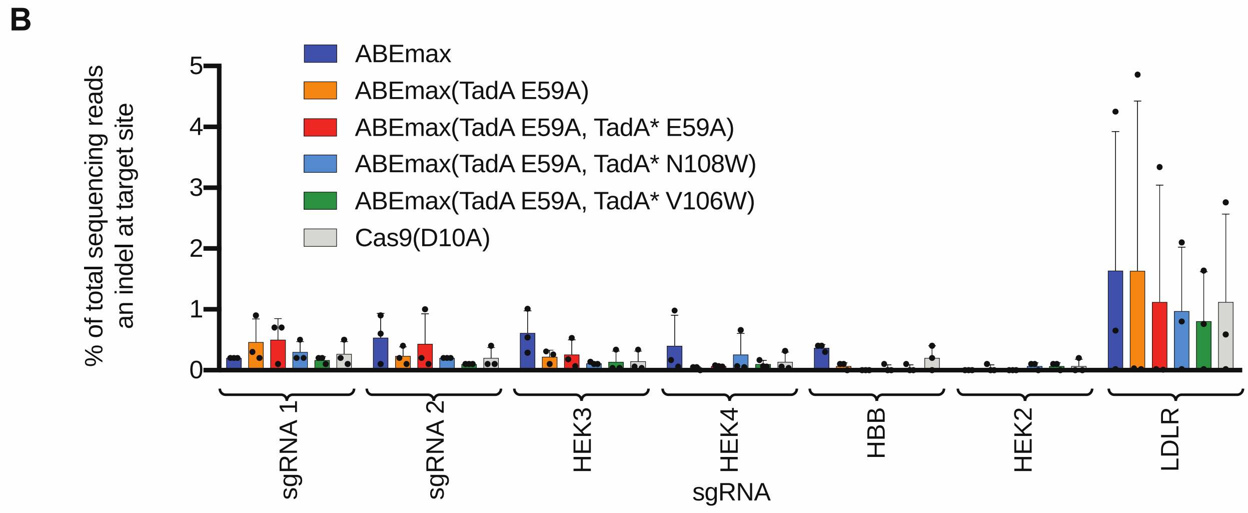

Fig. S2. DNA base editing and indel formation in HeLa cells from ABEmax and ABEmax mutants. To measure DNA base editing **(A)** and indel formation **(B)**, HeLa cells were lipofected with the indicated base editor plasmid combined with the indicated sgRNA plasmid. After 48 h, genomic DNA was harvested, amplified by PCR, and subjected to HTS. Data are shown as individual data points and mean±s.d. for n=3 independent biological replicates performed at different times

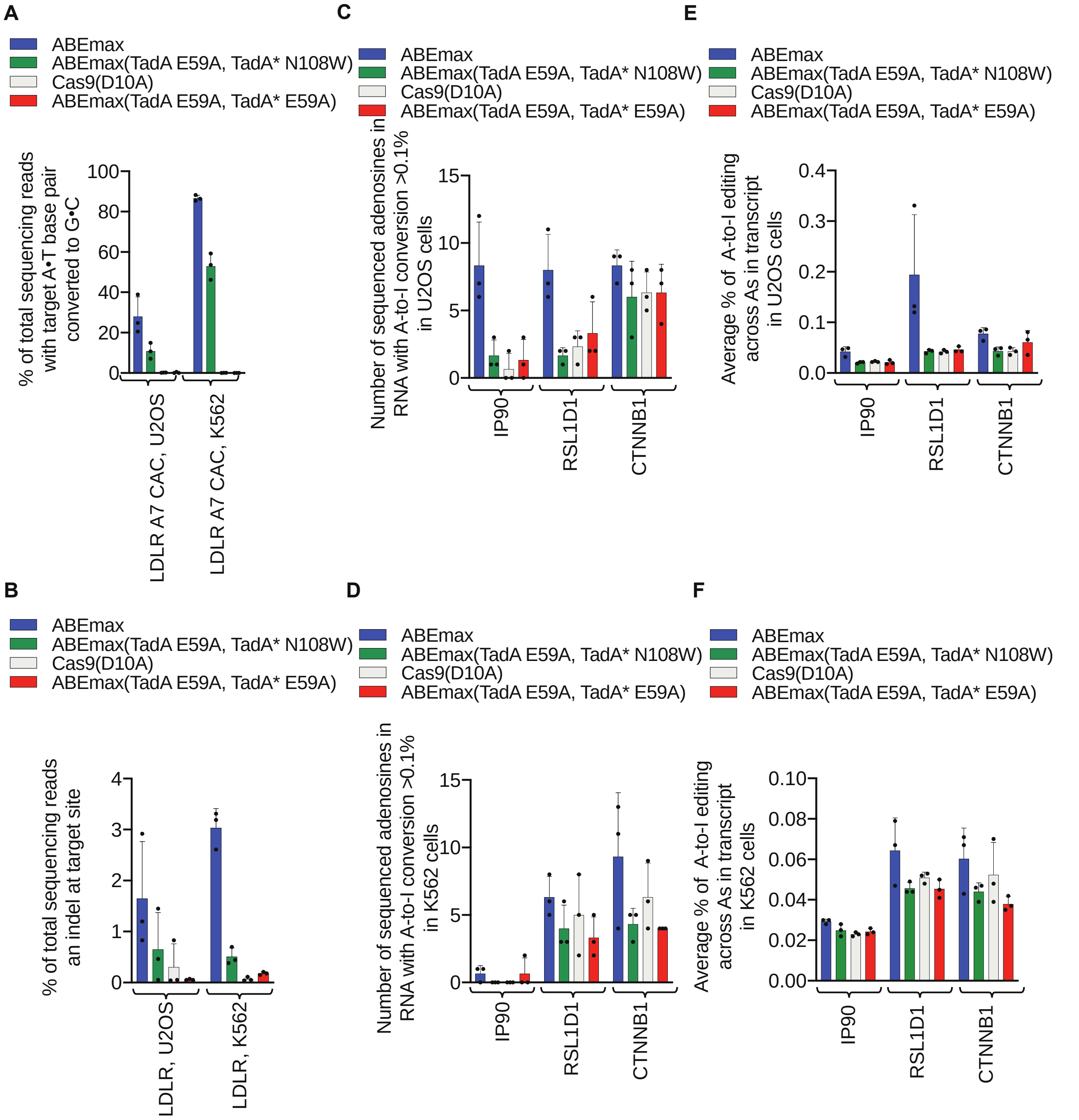

Fig. S3. DNA base editing, indel formation, and RNA editing in U2OS and K562 cells harvested 48 hours after nucleofection with ABEmax, ABEmax mutants, or Cas9(D10A). (A) DNA base editing efficiencies and **(B)** indel frequencies were measured in indicated cells 48 h days after nucleofection by HTS. RNA from nucleofected U2OS or K562 cells was harvested simultaneously with genomic DNA, and reverse transcription and HTS were used to assess **(C)** the frequency of sequenced adenosines in three mRNA transcripts with measurable A-to-I conversion in U2OS cells, **(D)** the average frequency of A-to-I conversion in three mRNA transcripts in U2OS cells, **(E)** the frequency of sequenced adenosines in three mRNA transcripts with measurable A-to-I conversion in K562 cells, **(F)** the average frequency of A-to-I conversion in three mRNA transcripts in K562 cells. Data are shown as individual data points and mean±s.d. for n=3 independent biological replicates.

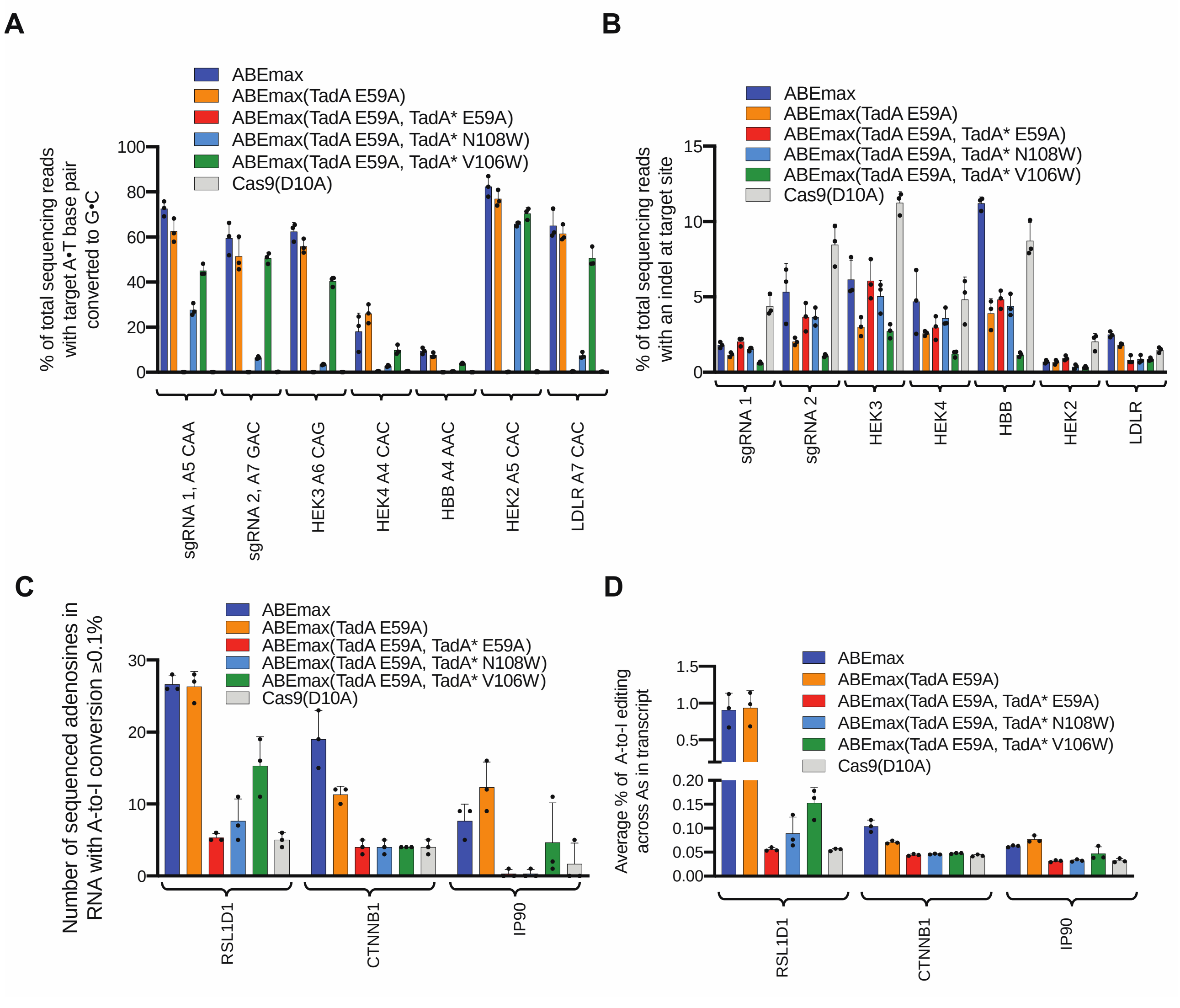

Fig. S4. DNA base editing, indel formation, and RNA editing in HEK293T cells harvested 5 days after transfection with ABEmax or ABEmax mutants. (A) DNA base editing efficiencies and **(B)** indel frequencies were measured in HEK293T cells 5 days after transfection. RNA from transfected HEK293T cells was harvested simultaneously with genomic DNA, and reverse transcription and HTS was used to assess **(C)** the frequency of sequenced adenosines with measurable A-to-I conversion and **(D)** the average frequency of A-to-I conversion in three mRNA transcripts. Data are shown as individual data points and mean±s.d. for n=3 independent biological replicates performed at different times.

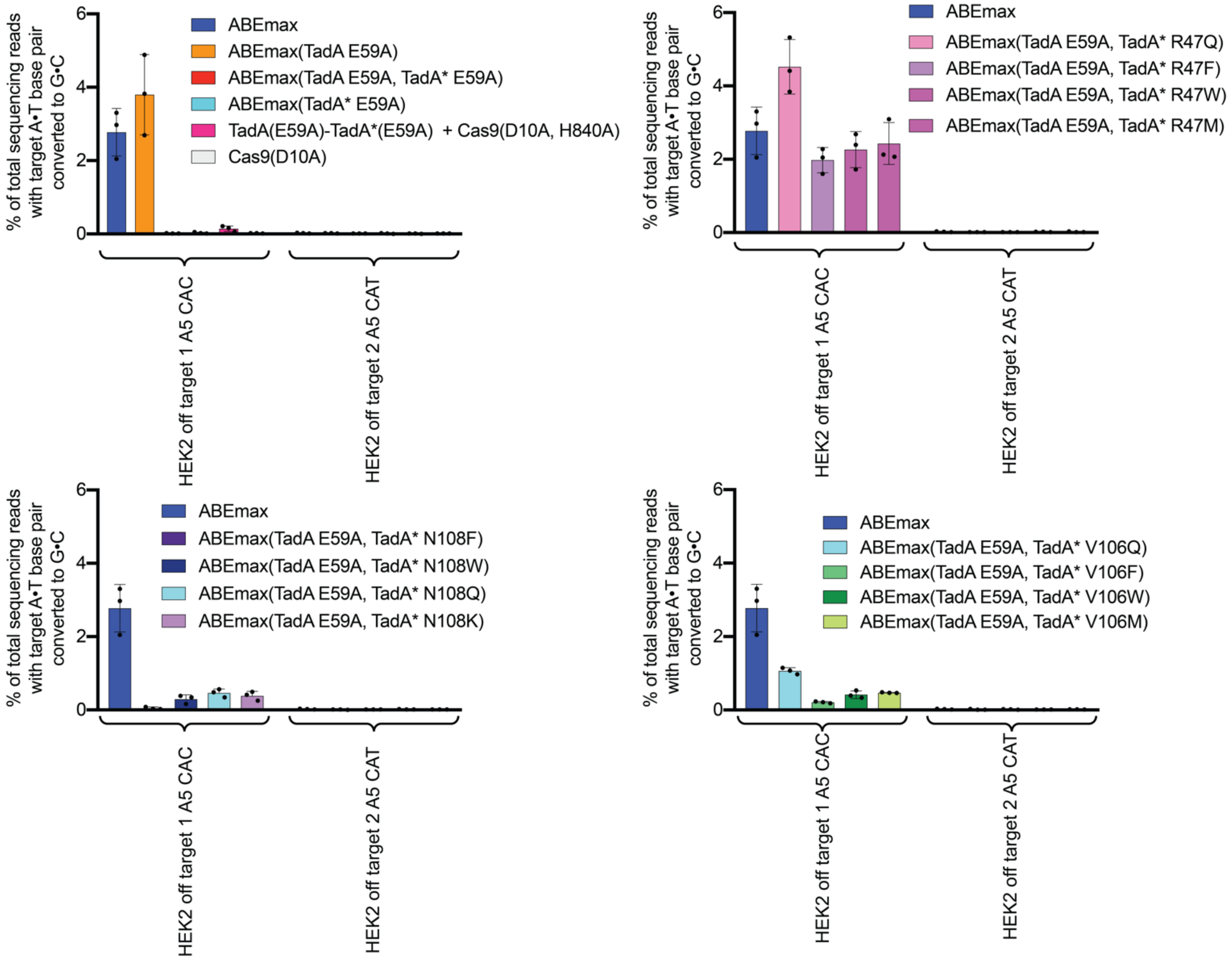

Fig. S5. Off-target DNA base editing associated with the HEK site 2 locus by ABEmax and ABEmax mutants. Off-target genomic DNA loci for the HEK site 2 sgRNA previously identified by GUIDE-Seq (*28*) were analyzed by HTS following treatment with the indicated ABEmax variants. Data are shown as individual data points and mean±s.d. for n=3 independent biological replicates, performed on different days.

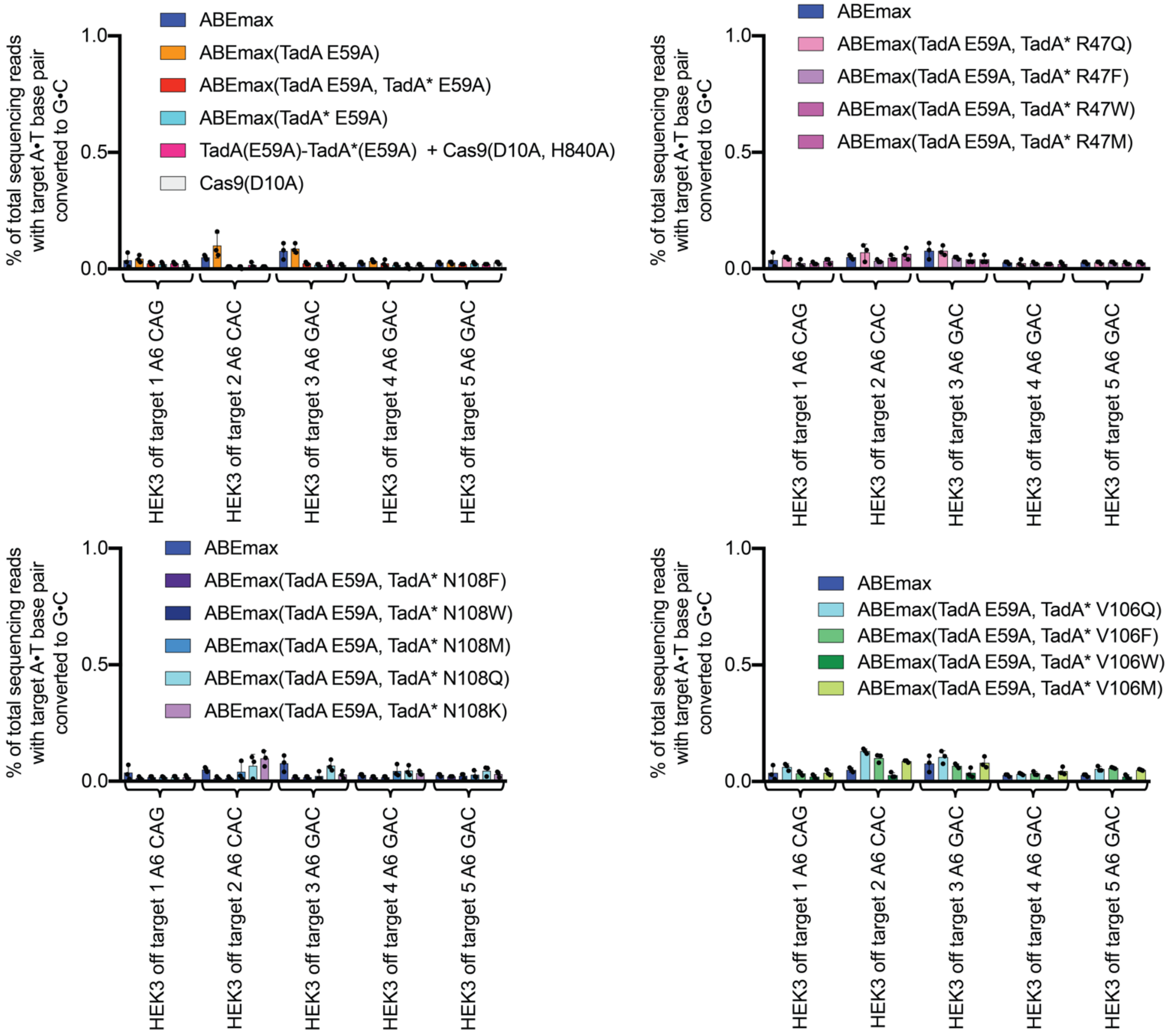

Fig. S6. Off-target DNA base editing associated with the HEK site 3 locus by ABEmax and ABEmax mutants. Off-target genomic DNA loci for the HEK site 3 sgRNA previously identified by GUIDE-Seq (*28*) were analyzed by HTS following treatment with the indicated ABEmax variants. Data are shown as individual data points and mean±s.d. for n=3 independent biological replicates, performed on different days.

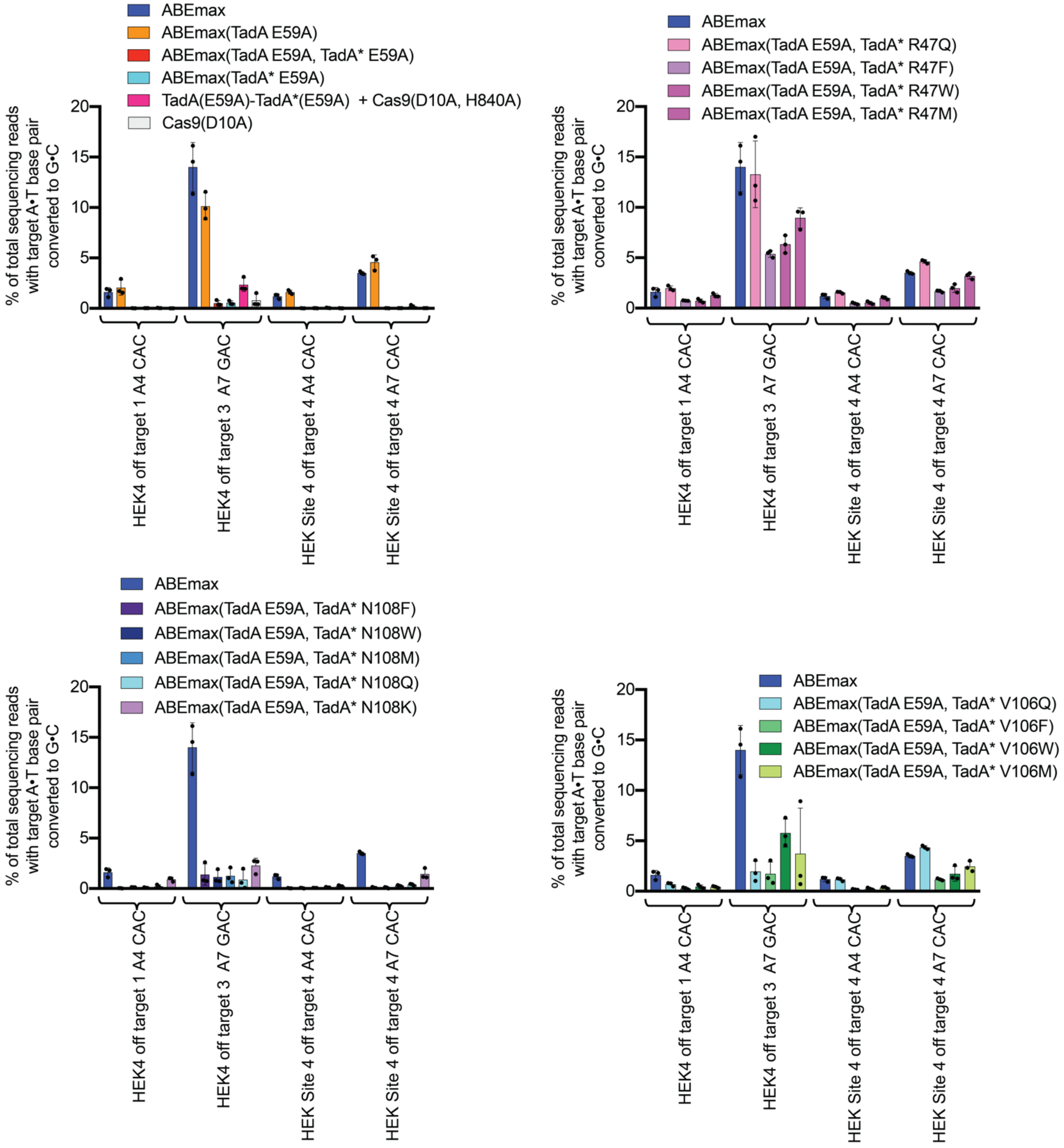

Fig. S7. Off-target DNA base editing associated with the HEK site 4 locus by ABEmax and ABEmax mutants. Off-target genomic DNA loci for the HEK site 4 sgRNA previously identified by GUIDE-Seq (*28*) were analyzed by HTS following treatment with the indicated ABEmax variants. Data are shown as individual data points and mean±s.d. for n=3 independent biological replicates, performed on different days.

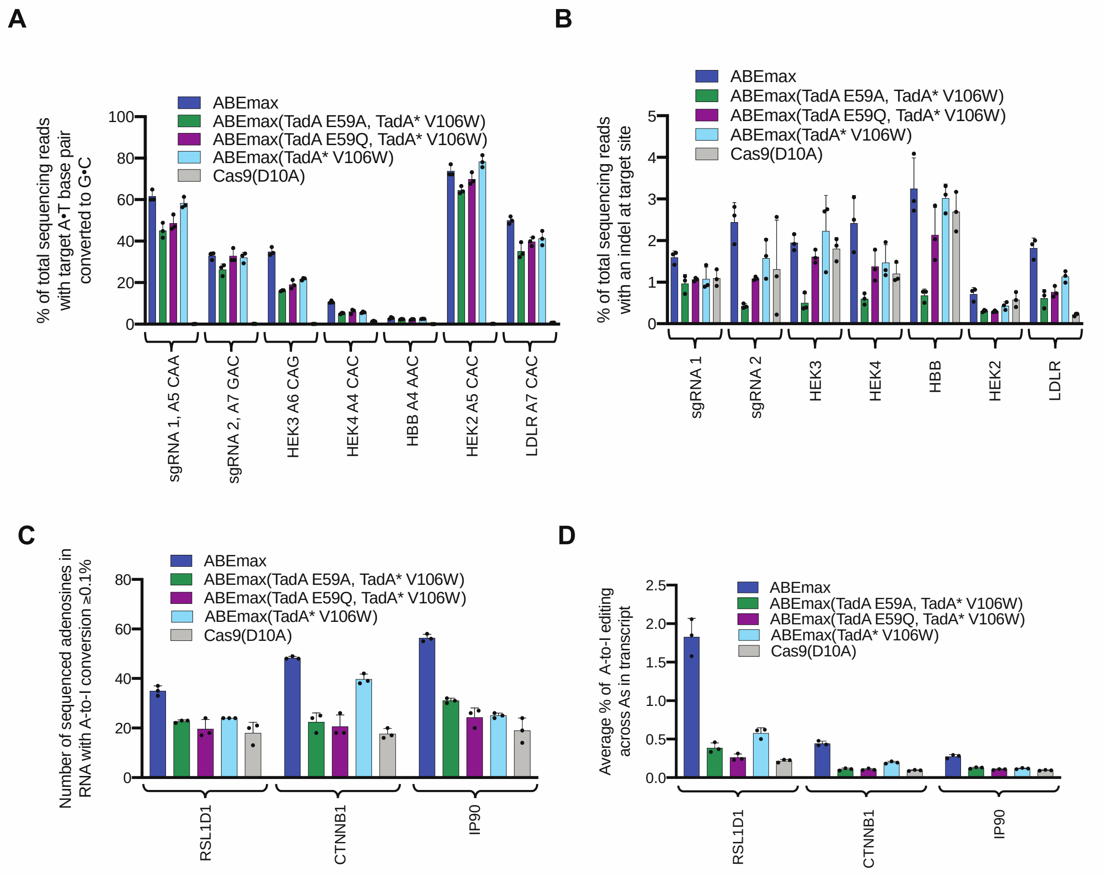

Fig. S8. DNA base editing, indel formation, and RNA editing in HEK293T cells harvested 48 hours after transfection with ABEmax, ABEmaxAW, ABEmaxQW or ABEmax(TadA* A106V). (A) DNA base editing efficiencies and **(B)** indel frequencies were measured in HEK293T cells harvested 48h after transfection. RNA from transfected HEK293T cells was harvested simultaneously with genomic DNA, and reverse transcription and HTS was used to assess **(C)** the frequency of sequenced adenosines with measurable A-to-I conversion and **(D)** the average frequency of A-to-I conversion in three mRNA transcripts. Data are shown as individual data points and mean±s.d. for n=3 independent biological replicates performed at different times.

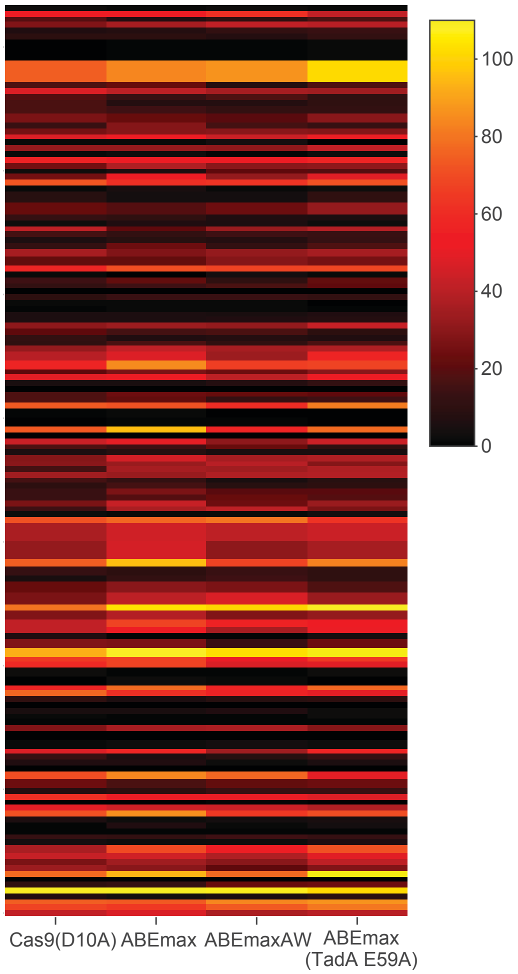

Fig. S9. A-to-I RNA editing across the transcriptome for ABEmax, ABEmaxAW, ABEmax(TadA E59A), and Cas9(D10A). A-to-I variant calls were plotted by transcript location. Bins 1,000,000 nucleotides wide are represented by each colored band. The number of high confidence A-to-I edits per bin are plotted to show the density of A-to-I edits per bin.

A

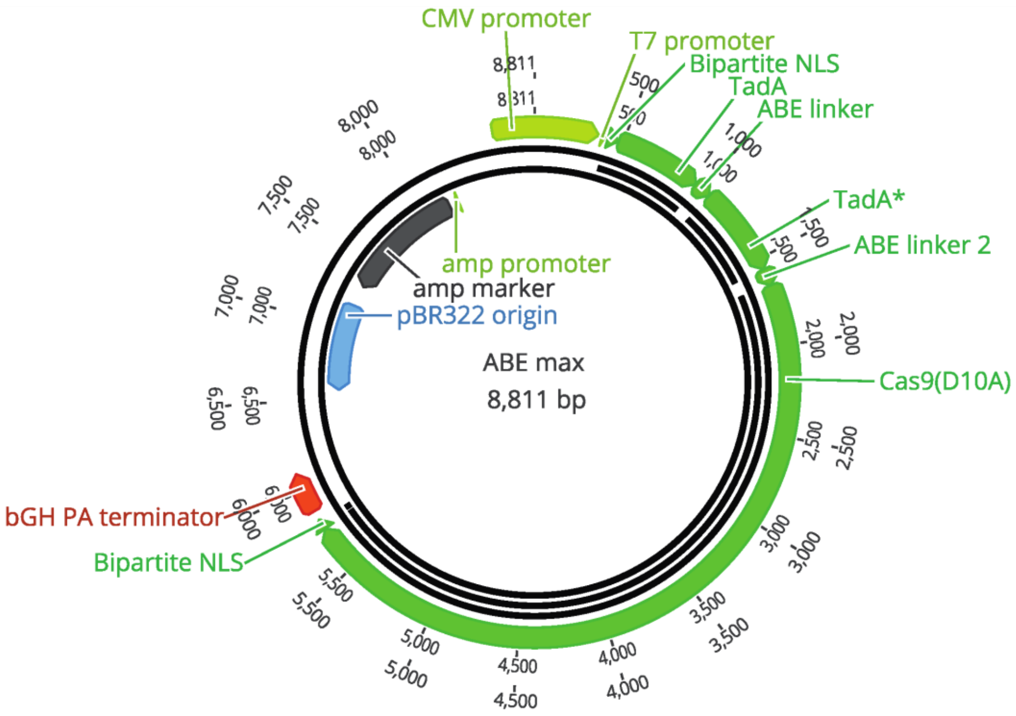

B

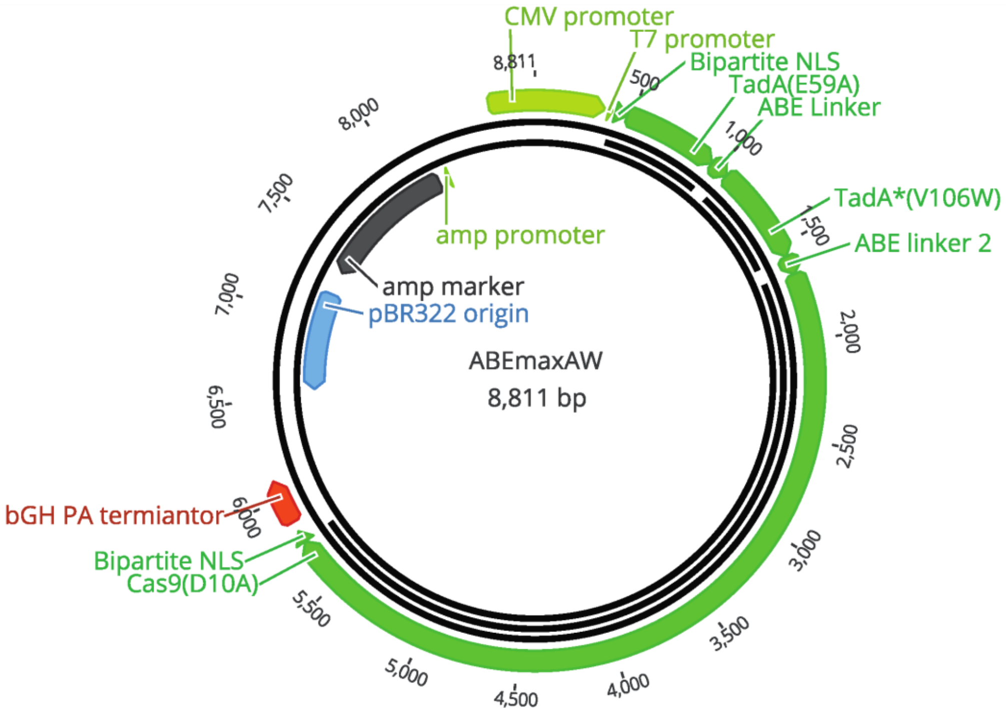

Fig. S10. Depiction of plasmid maps used in this study. The architecture of (**A**) ABEmax and (**B**) ABEmaxAW are shown.

Table S1. Guide RNA sequences. PAM sequences are in italics. For sgRNA LDLR, a 5’ G was included in the sgRNA expression cassette to enable efficient expression of the sgRNA from the U6 promoter. This 5’ G is indicated as [G].

**sgRNA name**	**sgRNA + PAM sequence**
sgRNA 1	GAGCAAAGAGAATAGACTGT*AGG*
sgRNA 2	GGATTGACCCAGGCCAGGGC*TGG*
HEK2	GAACACAAAGCATAGACTGC*CGG*
HEK3	GGCCCAGACTGAGCACGTGA*TGG*
HEK4	GGCACTGCGGCTGGAGGTGG*GGG*
HBB	GTAACGGCAGACTTCTCCTC*AGG*
LDLR	[G]CAGAGCACTGGAATTCGTCA*GGG*

Table S2. Primers used for amplification of genomic DNA or cDNA for HTS.

Primers for amplification of genomic DNA or cDNA	
LDLR forward	ACACTCTTTCCCTACACGACGCTCTTCCGATCTNNNNGCCCTGCTTCTTTTTCTCTGGT
LDLR reverse	TGGAGTTCAGACGTGTGCTCTTCCGATCTACCATTAACGCAGCCAACTTCA
HBB forward	ACACTCTTTCCCTACACGACGCTCTTCCGATCTNNNNGTCTTCTCTGTCTCCACATGCC
HBB reverse	TGGAGTTCAGACGTGTGCTCTTCCGATCTTAGGGTTGGCCAATCTACTCCC
HEK site 3 and sgRNA 2 forward	ACACTCTTTCCCTACACGACGCTCTTCCGATCTNNNNGGAAACGCCCATGCAATTAGTC
HEK site 3 and sgRNA 2 reverse	TGGAGTTCAGACGTGTGCTCTTCCGATCTCTTGTCAACCAGTATCCCGGTG
HEK site 2 forward	ACACTCTTTCCCTACACGACGCTCTTCCGATCTNNNNTGAATGGATTCCTTGGAAACAATG
HEK site 2 reverse	TGGAGTTCAGACGTGTGCTCTTCCGATCTCCAGCCCCATCTGTCAAACT
HEK site 4 forward	TGGAGTTCAGACGTGTGCTCTTCCGATCTTCCTTTCAACCCGAACGGAG
HEK site 4 reverse	ACACTCTTTCCCTACACGACGCTCTTCCGATCTNNNNGCTGGTCTTCTTTCCCCTCC
sgRNA 1 forward	ACACTCTTTCCCTACACGACGCTCTTCCGATCTNNNNGAGTTACTGCTCAGACATGTAA
sgRNA 1 reverse	TGGAGTTCAGACGTGTGCTCTTCCGATCTGACCTCGTGATCCACCTGCC
CTNNB1 forward	ACACTCTTTCCCTACACGACGCTCTTCCGATCTNNNNATTTGATGGAGTTGGACATGGCC
CTNNB1 reverse	TGGAGTTCAGACGTGTGCTCTCCAGCTACTTGTTCTTGAGTGAAGG
RSL1D1 forward	ACACTCTTTCCCTACACGACGCTCTTCCGATCTNNNNTGGCTTTCCAAATCAGTGGGTC
RSL1D1 reverse	TGGAGTTCAGACGTGTGCTCTTCCGATCTCTCATAAGCTTAGACCAACAAGC
IP90 forward	ACACTCTTTCCCTACACGACGCTCTTCCGATCTNNNNCTGGTTGACCAATCTGTGGTG
IP90 reverse	TGGAGTTCAGACGTGTGCTCTCTGCGTCTGGATCAGGTACG

Table S3. List of amplicon sequences used for alignment and analysis of HTS reads.

**DNA or RNA site**	**Amplicon sequence**
HEK4	TGGTCTTCTTTCCCCTCCCCTGCCCTCCCCTCCCTTCAAGATGGCTGACAAAGGCCGGGCTGGGTGGAAGGAAGGGAGGAAGGGCGAGGCAGAGGGTCCAAAGCAGGATGACAGGCAGGGGCACCGCGGCGCCCCGGTGGCACTGCGGCTGGAGGTGGGGGTTAAAGCGGAGACTCTGGTGCTGTGTGACTACAGTGGGGGCCCTGCCCTCTCTGAGCCCCCGCCTCCAGGCCTGTGTGTGTGT
HEK3	GGAAACGCCCATGCAATTAGTCTATTTCTGCTGCAAGTAAGCATGCATTTGTAGGCTTGATGCTTTTTTTCTGCTTCTCCAGCCCTGGCCTGGGTCAATCCTTGGGGCCCAGACTGAGCACGTGATGGCAGAGGAAAGGAAGCCCTGCTTCCTCCAGAGGGCGTCGCAGGACAGCTTTTCCTAGACAGGGGCTAGTATGTGCAGCTCCTGCACCGGGATACTGGTTGACAAG
HEK2	TGAATGGATTCCTTGGAAACAATGATAACAAGACCTGGCTGAGCTAACTGTGACAGCATGTGGTAATTTTCCAGCCCGCTGGCCCTGTAAAGGAAACTGGAACACAAAGCATAGACTGCGGGGCGGGCCAGCCTGAATAGCTGCAAACAAGTGCAGAATATCTGATGATGTCATACGCACAGTTTGACAGATGGGGCTGG
LDLR	GCCCTGCTTCTTTTTCTCTGGTTGTCTCTTCTTGAGAAAATCAACACACTCTGTCCTGTTTTCCAGCTGTGGCCACCTGTCGCCCTGACGAATTCCAGTGCTCTGATGGAAACTGCATCCATGGCAGCCGGCAGTGTGACCGGGAATATGACTGCAAGGACATGAGCGATGAAGTTGGCTGCGTTAATGGT
HBB	CTTCTCTGTCTCCACATGCCCAGTTTCTATTGGTCTCCTTAAACCTGTCTTGTAACCTTGATACCAACCTGCCCAGGGCCTCACCACCAACTTCATCCACGTTCACCTTGCCCCACAGGGCAGTAACGGCAGACTTCTCCTCAGGAGTCAGATGCACCATGGTGTCTGTTTGAGGTTGCTAGTGAACACAGTTGTGTCAGAAGCAAATGTAAGCAATAGATGGCTCTGCCCTGACTTTTATGCCCAGCCCTGGCTCCTGCCCTCCCTGCTCCTGGGAGTAGATTGGCCAA
sgRNA 1	GTTACTGCTCAGACATGTAATAATAATAAATAACACATCAAATAACCATACCATTTTAAGCTGTAGTATTATGAAGGGAAATCTGGAGCAAAGAGAATAGACTGTAGGGAAACCAGTTAAGAAATAGGACATGGAGGCTAGGTGCAGTGGCTCACGCCTGTAATCGCAGCACTTTGGGAGGCTGAGGCAGGTGGATCACGAGG
sgRNA 2	GGAAACGCCCATGCAATTAGTCTATTTCTGCTGCAAGTAAGCATGCATTTGTAGGCTTGATGCTTTTTTTCTGCTTCTCCAGCCCTGGCCTGGGTCAATCCTTGGGGCCCAGACTGAGCACGTGATGGCAGAGGAAAGGAAGCCCTGCTTCCTCCAGAGGGCGTCGCAGGACAGCTTTTCCTAGACAGGGGCTAGTATGTGCAGCTCCTGCACCGGGATACTGGTTGACAAG
RSL1D1	TTGGCTTTCCAAATCAGTGGGTCTGACTTGAGGTCTGTGATGTGACCCTTTTCCTCACCTGCTCAACCATTATTCACATGGACTCCATCATATTCATTTGTAGTCATTCCCAGAGTGGCCCAGTGAGGGTCTCGCTGTATGAGAGTCGGCTACGGAATTTAGGAGAAACAGAAGTTTCTTGGCTTTCATGCTGAGCTTGTTGGTCTAAGCTTATGAG
CTNNB1	TTTGATGGAGTTGGACATGGCCATGGAACCAGACAGAAAAGCGGCTGTTAGTCACTGGCAGCAACAGTCTTACCTGGACTCTGGAATCCATTCTGGTGCCACTACCACAGCTCCTTCTCTGAGTGGTAAAGGCAATCCTGAGGAAGAGGATGTGGATACCTCCCAAGTCCTGTATGAGTGGGAACAGGGATTTTCTCAGTCCTTCACTCAAGAACAAGTAGCTGG
IP90	CTGGTTGACCAATCTGTGGTGAATAGTGGAAATCTGCTCAATGACATGACTCCTCCTGTAAATCCTTCACGTGAAATTGAGGACCCAGAAGACCGGAAGCCCGAGGATTGGGATGAAAGACCAAAAATCCCAGATCCAGAAGCTGTCAAGCCAGATGACTGGGATGAAGATGCCCCTGCTAAGATTCCAGATGAAGAGGCCACAAAACCCGAAGGCTGGTTAGATGATGAGCCTGAGTACGTAC

Table S4. List of primers used to amplify genomic off-target loci. These primers have been published previously (*1, 2*) but are listed here for completeness.

Target site	Primer sequence
forward HEK site2 off1	ACACTCTTTCCCTACACGACGCTCTTCCGATCTNNNNGTGTGGAGAGTGAGTAAGCCA
reverse HEK site2 off1	TGGAGTTCAGACGTGTGCTCTTCCGATCTACGGTAGGATGATTTCAGGCA
forward HEK site2 off2	ACACTCTTTCCCTACACGACGCTCTTCCGATCTNNNNCACAAAGCAGTGTAGCTCAGG
reverse HEK site2 off2	TGGAGTTCAGACGTGTGCTCTTCCGATCTTTTTTGGTACTCGAGTGTTATTCAG
forward HEK site3 off1	ACACTCTTTCCCTACACGACGCTCTTCCGATCTNNNNTCCCCTGTTGACCTGGAGAA
reverse HEK site3 off1	TGGAGTTCAGACGTGTGCTCTTCCGATCTCACTGTACTTGCCCTGACCA
forward HEK site3 off2	ACACTCTTTCCCTACACGACGCTCTTCCGATCTNNNNTTGGTGTTGACAGGGAGCAA
reverse HEK site3 off2	TGGAGTTCAGACGTGTGCTCTTCCGATCTCTGAGATGTGGGCAGAAGGG
forward HEK site3 off3	ACACTCTTTCCCTACACGACGCTCTTCCGATCTNNNNTGAGAGGGAACAGAAGGGCT
reverse HEK site3 off3	TGGAGTTCAGACGTGTGCTCTTCCGATCTGTCCAAAGGCCCAAGAACCT
forward HEK site3 off4	ACACTCTTTCCCTACACGACGCTCTTCCGATCTNNNNTCCTAGCACTTTGGAAGGTCG
reverse HEK site3 off4	TGGAGTTCAGACGTGTGCTCTTCCGATCTGCTCATCTTAATCTGCTCAGCC
forward HEK site3 off5	ACACTCTTTCCCTACACGACGCTCTTCCGATCTNNNNAAAGGAGCAGCTCTTCCTGG
reverse HEK site3 off5	TGGAGTTCAGACGTGTGCTCTTCCGATCTGTCTGCACCATCTCCCACAA
forward HEK site4 off1	ACACTCTTTCCCTACACGACGCTCTTCCGATCTNNNNGGCATGGCTTCTGAGACTCA
reverse HEK site4 off1	TGGAGTTCAGACGTGTGCTCTTCCGATCTGTCTCCCTTGCACTCCCTGTCTTT
forward HEK site4 off2	ACACTCTTTCCCTACACGACGCTCTTCCGATCTNNNNTTTGGCAATGGAGGCATTGG
reverse HEK site4 off2	TGGAGTTCAGACGTGTGCTCTTCCGATCTGAAGAGGCTGCCCATGAGAG
forward HEK site4 off3	ACACTCTTTCCCTACACGACGCTCTTCCGATCTNNNNGGTCTGAGGCTCGAATCCTG
reverse HEK site4 off3	TGGAGTTCAGACGTGTGCTCTTCCGATCTCTGTGGCCTCCATATCCCTG
forward HEK site4 off4	ACACTCTTTCCCTACACGACGCTCTTCCGATCTNNNNTTTCCACCAGAACTCAGCCC
reverse HEK site4 off4	TGGAGTTCAGACGTGTGCTCTTCCGATCTCCTCGGTTCCTCCACAACAC
forward HEK site4 off5	ACACTCTTTCCCTACACGACGCTCTTCCGATCTNNNNCACGGGAAGGACAGGAGAAG
reverse HEK site4 off5	TGGAGTTCAGACGTGTGCTCTTCCGATCTGCAGGGGAGGGATAAAGCAG

Table S5. List of interrogated off-target genomic loci (*28*), with guide RNA sequences and amplicons used for alignment.

Name	sgRNA sequence	Amplicon sequence
HEK site2 off1	GAACACAATG CATAGATTGC	GTGTGGAGAGTGAGTAAGCCAGAACACAATGCATAGATTGCCGGTAAATAGGTTTA GATTCATCCATTTTTAAAAAATGGTGTGGGAGCATTAAATATGTATATAGTAGATATG GAAAAATGATTCTCATAATAACTGACATTTCTGTTTCACAAGAAAATTATTTTACATTA TATGTATATTTTACATAAATTATACATAGTCATTTAAAAAGCTCAAATAGTGCAAAAAC AATATGGAGAATTGCCTGAAATCATCCTACCGT
HEK site2 off2	AAACATAAAGC ATAGACTGC	CACAAAGCAGTGTAGCTCAGGGAAGGAGCAGTGAGTTTGGGCACTTGTGACAGAAT AGTGGGACTATGCCAGAGATACACAGGAGGAGGTGGTACCTTCTAGCTCCCCCTCA AAACATAAAGCATAGACTGCAAAGTACTCCCAAGCAGGCTGAATAACACTCGAGTA CCAAAAA
HEK site3 off1	CACCCAGACT GAGCACGTGC	TCCCCTGTTGACCTGGAGAAGCATGAACCAGTCAAAAAGTTTAAAGACAAGAGCATT AACTGCACCAGTGGGCAGCTCAGCTCAGACACCAGTAGCGTGGGCACCCAGACTG AGCACGTGCTGGAGCCCAAGAAATGCAGAGACCTGTGCACCTCTGGTCAGGGCAA GTACAGTG
HEK site3 off2	GACACAGACC GGGCACGTGA	TTGGTGTTGACAGGGAGCAACTTCACAGTCCCAGGCATCAGGACACAGACCGGGC ACGTGAGGGAAGCCCAAGGGAGAGGACTGGTGTAATCGAGGCTGACTCCACTTTT AATGTTTGACTGATGATAGGTTTCAAGTCTCACTAAGTCTCCTTCCCCTTCTGCCCA CATCTCAG
HEK site3 off3	AGCTCAGACT GAGCAAGTGA	TGAGAGGGAACAGAAGGGCTAAGACTAAAAGGAACAGAGGAGTTCATAGTGAGCG GTAAAGAGCTCAGACTGAGCAAGTGAGGGGCTCAGCCTCCCATGGAGGACAGGGG GCTGGGGCCCCTGGCTGATGTCTGGACTGAAGCCCCCACGCCCAGAGGTTCTTGG GCCTTTGGAC
HEK site3 off4	AGACCAGACT GAGCAAGAGA	CCTAGCACTTTGGAAGGTCGAAGCGGCAGGATGGCTTCAACCCAGGAGTTCGAGA CCAGACTGAGCAAGAGAGGGAGAGTGTCTGTATTAACAACAAACAAACAAACAAAA AACTAAACTAAAAGAAACTGTGGTGTATAATATAAAATTCTGGCTGAGCAGATTAAG ATGAGC
HEK site3 off5	GAGCCAGAAT GAGCACGTGA	AAAGGAGCAGCTCTTCCTGGTGGAAATTGCGAGCAGAGGCTGCGTGAGTTCCGTA ACTCGCACACAGCCTCCATTTGGAGCCAGAATGAGCACGTGAGGGACCCCGGGCA GAGGGGCCAGTGCTGACATTATGCTCCATGCAACCTCCCATCCTGTTGTGGGAGAT GGTGCAGAC
HEK site2 off1	TGCACTGCGG CCGGAGGAGG	GGCATGGCTTCTGAGACTCATAGCTGGGGCTGAAGATCCCTAGGGGGGCTCTGCT GGGCTCACTGCTCTCCAGAGTGGTCCAGCCCGGCTGCAGGGTGCTGCTTCCAGCT TGGTGCACTGCGGCCGGAGGAGGTGGAGGATGGAAAGTAAGATTCAAAGACAGGG AGTGCAAGGG
HEK site2 off2	GGCTCTGCGG CTGGAGGGGG	TTTGGCAATGGAGGCATTGGGCAGGGGAAGCCTGTCTTCAGGGCACATGCACGTG CGCAGGGCTCTGCGGCTGGAGGGGGTGGGGTTGCTGTTAGTGACAGGGGCCCCA GCCAGGCAGGTTTCAGGATTGGGGAGCACTTGCTTCGGCTCCCTTGCTCTCATGG GCAGCCTCTTC
HEK site2 off3	GGCACGACGG CTGGAGGTGG	GGTCTGAGGCTCGAATCCTGGCAGCAGGTCCTTCATGGCAAGGCGGGAAAAGAGA AAAGCCAACGGGTTCTCATGCTGGGAAAAGATGCCGGGCACGACGGCTGGAGGTG GGGGGTTGGGAGTGGGTGGGATGCTTGCGTGCCCTGCATGAGGTGCAGGGATAT GGAGGCCACAG
HEK site2 off4	GGCATCACGG CTGGAGGTGG	TTCCACCAGAACTCAGCCCAGGCTGCTGTGGGATGGAATCACCTGCACCCGGATG TTCTTTCTGGGCTGGTACATACAGGCAAGGCATCACGGCTGGAGGTGGAGGGGGC CTAACCCGGGGTTGCCCAGGAAGGGGTTTGCACATGGATTCGGTGTGTTGTGGAG GAACCGAGG
HEK site2 off5	GGCGCTGCGG CGGGAGGTGG	CACGGGAAGGACAGGAGAAGGTGCTGGACCGCCTGGACTTTGTGCTGACCAGCCT TGTGGCGCTGCGGCGGGAGGTGGAGGAGCTGAGAAGCAGCCTGCGAGGGCTTGC GGGGGAGATTGTTGGGGAGGTCCGGTGAGTAATGCGGCTTCTTCTCCTGCTTTATC CCTCCCCTGC

Table S6. List of plasmid accession numbers from Addgene.

Plasmid name	Addgene number
pCMV-TadA-TadA*	125661
pCMV-ABEmax(TadA E59A, TadA*R47M)	125660
pCMV-ABEmax(TadA E59A, TadA*R47W)	125659
pCMV-ABEmax(TadA E59A, TadA*R47F)	125658
pCMV-ABEmax(TadA E59A, TadA*R47Q)	125657
pCMV-ABEmax(TadA E59A, TadA*D108M)	125656
pCMV-ABEmax(TadA E59A, TadA*D108Q)	125655
pCMV-ABEmax(TadA E59A, TadA*D108F)	125654
pCMV-ABEmax(TadA E59A, TadA*D108W)	125653
pCMV-ABEmax(TadA E59A, TadA*V106F)	125652
pCMV-ABEmax(TadA E59A, TadA*V106Q)	125651
pCMV-ABEmax(TadA E59A, TadA*V106M)	125650
pCMV-ABEmax(TadA E59A, TadA*E59A)	125649
pCMV-ABEmax(TadA E59A)	125648
pCMV-ABEmaxAW	125647
pCMV-ABEmax(TadA, TadA*E59A)	125662
